# Parents’/caregivers’ fears and concerns about their child’s epilepsy: A scoping review

**DOI:** 10.1371/journal.pone.0274001

**Published:** 2022-09-06

**Authors:** Bernie Carter, Georgia Cook, Lucy Bray, Amber Collingwood, Holly Saron, Alison Rouncefield-Swales

**Affiliations:** 1 Faculty of Health, Social Care and Medicine, Edge Hill University, Ormskirk, United Kingdom; 2 Faculty of Health and Life Sciences, Centre for Psychological Research, Department of Psychology, Health and Professional Development, Oxford Brookes University, Oxford, United Kingdom; 3 Department of Basic and Clinical Neuroscience, Institute of Psychiatry, Psychology and Neuroscience, King’s College London, London, United Kingdom; 4 Allied Health Research Unit, University of Central Lancashire, Preston, Lancashire, United Kingdom; Xiamen University - Malaysia Campus: Xiamen University - Malaysia, MALAYSIA

## Abstract

**Background:**

Childhood epilepsy is a serious and common neurological condition and can have life-long consequences and its impact can pervade all aspects of family life. Whilst the medical management of seizures is important, much of the day-to-day home management of epilepsy is invisible to people external to the family, including health care professionals, and parents’/caregivers’ fears and concerns can go unacknowledged and unaddressed by health care professionals.

**Objective:**

This objective of this review was to examine parents’/caregivers’ fears and concerns regarding their child’s epilepsy, the impact of these fears and concerns on family life, the social and emotional well-being of parents/caregivers and any factors which mitigate these fears and concerns.

**Design:**

Scoping review using a modified version of Arksey and O’Malley’s framework.

**Data sources:**

Relevant studies were identified using key search terms in Scopus, Medline, CINAHL and PsychInfo databases in March 2021 with hand checking of reference lists. Search terms were developed using population (parents/caregivers of children aged ≤ 18 years with epilepsy, families); concept (parents’/caregivers’ fears, concerns, anxiety about their child’s epilepsy); and context (any setting). A further search was run in April 2022. Other inclusion criteria: English language empirical studies, 2010–2021.

**Study appraisal methods:**

A minimum of two reviewers independently screened articles and undertook data extraction and decisions were consensually made.

Methodological quality appraisal was undertaken using the Mixed Methods Appraisal Tool v2018. A data extraction table was created to chart all studies. The conduct and reporting of this study followed the Preferred Reporting Items for Systematic Reviews and Meta-Analyses (PRISMA) guidance for Systematic reviews and Meta-Analyses extension for Scoping Reviews (PRISMA-ScR) (S1 Table). There is no published copy of the review protocol.

**Main findings:**

The search identified a total of 4077 papers (after duplicates were removed) of which 110 were assessed for eligibility. Twenty-four papers published between 2010–2021 were included in the review and each paper was treated as a separate study. The review findings indicate that parents’/caregivers’ fears and concerns stem from more than their child’s seizures and relate to many wider aspects of family life. These fears and concerns had far-reaching influences on their parenting/caregiving, and on the lifestyle and activities of their child and their family. What was less evident was what parents/caregivers wanted in terms of support or how they thought health professionals could acknowledge and/or allay their fears and concerns. The discussion is framed within the compassion-focused therapy model as a basis for generating new thinking about the impact of these fears and concerns and the need for a new agenda for clinical consultations in childhood epilepsy.

**Conclusions:**

The review concludes with a proposal that a more compassionate agenda underpins the dialogue between parents/caregivers and clinicians to encompass and mitigate the wider emotional, psychosocial, and societal threats that impact on the parent/caregivers of children with epilepsy.

## Background

Childhood epilepsy is a serious and common neurological condition and can have life-long consequences [1] and is the most common chronic neurological condition in childhood [2]. The reports of incidence vary with a global variation ranging from 41-187/100,000 with the highest incidence in underdeveloped countries [3]. Incidence also varies across age with early-onset epilepsy in children aged <60 months estimated to be 57–130 per 100,000 per year [4].

Evidence shows that a diagnosis of childhood epilepsy can have huge and often adverse influences on children and their parent/caregiver and on the family [5] with the impact reported as pervading all aspects of a family’s life [6], including school and education [7], outdoor recreation [8], social activities [9], interaction with health services [8] and sleep [10]. Children with epilepsy and their parents have reported increased levels of stress, anxiety and depression [11–13], lower quality of life [14] and higher levels of stigma [15]. Whilst the management of seizures is important, the evidence shows that much of the management of epilepsy is invisible [16] and the management of childhood epilepsy needs to be holistic and consider more than just seizure management as core elements of living with epilepsy [17]. Parents have reported a lack of support for these wider aspects of epilepsy care and that their fears and concerns, for example about their child’s sleep [18] can go unacknowledged and unaddressed by health care professionals. Whilst there has been some investigation of parents fears and concerns associated with caring for their child with a long-term condition [19], including diabetes [20] and asthma [21] there is less known about these in relation to being a parent of a child with epilepsy. Although studies and recent systematic reviews [6,13] have focussed on parents’ anxieties and fears associated with their child’s epilepsy. The intention of this review was to be more inclusive and go beyond the narrower focus adopted by the previous systematic reviews published in 2016 [6,13]. One review focused solely on qualitative research about families’ experiences of living with paediatric epilepsy [6] and the other focused on symptoms of anxiety reported by parents of children (0–18 years) with epilepsy and only included studies which used a standardized measure of anxiety or a measure for which psychometric data had been published [13]. Our review builds on these reviews by adopting a wider methodological inclusion and extending beyond anxiety to focus on parents’/caregivers’ fears about their child’s epilepsy as well as their potential concerns over their child’s future.

This scoping review will address the broad question: ‘What is known about parents’/caregivers’ fears and concerns regarding their child’s epilepsy and the impact of these fears and concerns on family life, as well as the social and emotional well-being of parents’/caregivers’ and any factors which mitigate these fears and concerns?’

## Method

A scoping review was undertaken as the intention was to explore and map the key concepts and identify gaps and discuss these concepts in research related to parents’/caregivers’ fears and concerns regarding their child’s epilepsy and through this process identify gaps in the current evidence. This broader discursive intention meant that a scoping review rather than a systematic review was appropriate as we were not intending to specifically answer a clinical question in terms of feasibility, meaningfulness or effectiveness [22].

The scoping review used a modified version of Arksey and O’Malley’s [23] framework to allow a more flexible and robust means of reporting our results. Our modifications included use of an iterative approach to refining our search strategy and inclusion criteria [24,25], and the use of the Mixed Methods Appraisal Tool v2018 (MMAT) [26] to assess methodological quality. The conduct and reporting of this study followed the Preferred Reporting Items for Systematic Reviews and Meta-Analyses (PRISMA) guidance for Systematic reviews and Meta-Analyses extension for Scoping Reviews (PRISMA-ScR) [27] (S1 Table). There is no published copy of the review protocol.

### Stage 1: Identifying the research question/objective

An overarching research question guided our systematic search strategy and reporting of results:

What is known from the existing literature about parents’/caregivers’ fears and concerns regarding their child’s epilepsy, their perceptions of the impact of their fears and concerns on family life, and on their own social and emotional well-being and what mitigates these fears and concerns? This question enabled us to adequately capture a broad range of existing literature while providing the opportunity for further research objectives to be added and modified throughout the review. This iterative process was useful as we became increasingly familiar with the literature although we did not need to amend the objectives.

### Stage 2: Identifying relevant studies

Our definition of fears and concerns was broad and encompassed anything reported as a fear or concern within the papers; this included terms such as worry, anxiety, psychological distress, and hypervigilance.

Search terms were developed based on consideration of the population (parents/caregivers of children aged ≤ 18 years with epilepsy, families); concept (parents’/caregivers’ fears, concerns, anxiety about their child’s epilepsy); and context (any setting) [28] (S2 Table). Keywords and terms were identified by the authors, other members of the CASTLE (Changing Agendas on Sleep, Treatment and Learning in Childhood Epilepsy) research study team and parents from the study’s Family Advisory Group (castlestudy.org.uk).

A comprehensive list of search terms was identified and refined through searches on Scopus and Medline by the review team. AR and GC led the development of the search strategy with the support of a health research librarian. Truncation and proximity operators were employed to increase the sensitivity of the search (S3 Table). Searches were undertaken in Scopus, Medline, CINAHL and PsychInfo databases in March 2021 (S4 Table).

The reference lists of included papers were reviewed for additional papers, and Scopus and Google Scholar were consulted to identify the citing literature. A search of the grey literature (reports and webpages), including a hand search, was also completed in March 2021 (S4 Table). Grey literature was identified using similar search terms to those used in the main search via Open Grey, Google Scholar and from the websites of NICE Evidence search, Royal College of Paediatrics and Child Health, Royal College of Nursing, National Institute of Health Research portfolio, Department of Health, Epilepsy Action, Epilepsy Society, Epilepsy Research UK, Young Epilepsy, Epilepsy 12, International Bureau for Epilepsy (IBE), International League Against Epilepsy (ILAE), as well as other international epilepsy organisations. The Google Scholar search produced 7150 results, the first 520 were hand searched; this was a reasonable number of references to consider as recommendations are that for systematic review searches focus on the first 200–300 results [29].

### Stage 3: Study selection

Studies were included in the review if they met the criteria outlined in Table 1. The original search parameters were papers published 1st January 2010 – 23rd March 2021; it was updated on 15^th^ April 2022. The year range was limited to studies published from 2010 onwards as the focus was to explore relatively recent literature so that any recommendations for practice would be contemporary and meaningful. The parents/caregivers of children aged ≤ 18 years was used to encompass children and young people and was determined based partly on the World Health Organization (2014) [30] definition of a ‘child’ as a person under the age of 18 years but also reflecting that many studies of children include 18 year olds and we did not want to miss such studies from the review.

**Table 1:** Inclusion and exclusion criteria.

Inclusion criteria 1. Empirical studies (studies based on direct experience or observation of the world) 2. Parents/caregivers of children aged ≤ 18 years with epilepsy 3. Studies including parent/caregiver and family experiences of living with paediatric epilepsy 4. 2010–2021 (initial review) then to 15^th^ April 2022 (updated review) 5. English language papers
Exclusion criteria 1. Book reviews, opinion pieces, unpublished theses (as they would not necessarily have been subject to peer review, although the theses would have been subject to examination) 2. Systematic reviews 3. Studies that do not include parents/caregivers 4. Studies involving children with epilepsy and other major comorbid conditions but where the focus of the paper was not on epilepsy 5. Studies focused on the experience of transition from paediatric to adult care 6. Studies focused on epilepsy treatments such as surgical interventions 7. Studies focused solely on the incidence of HRQoL, Depression, Anxiety and Stress 8. Studies focused solely on the communication of SUDEP

### Inclusion criteria and types of sources

The inclusion and exclusion criteria are shown in Table 1.

**Fig 1:**
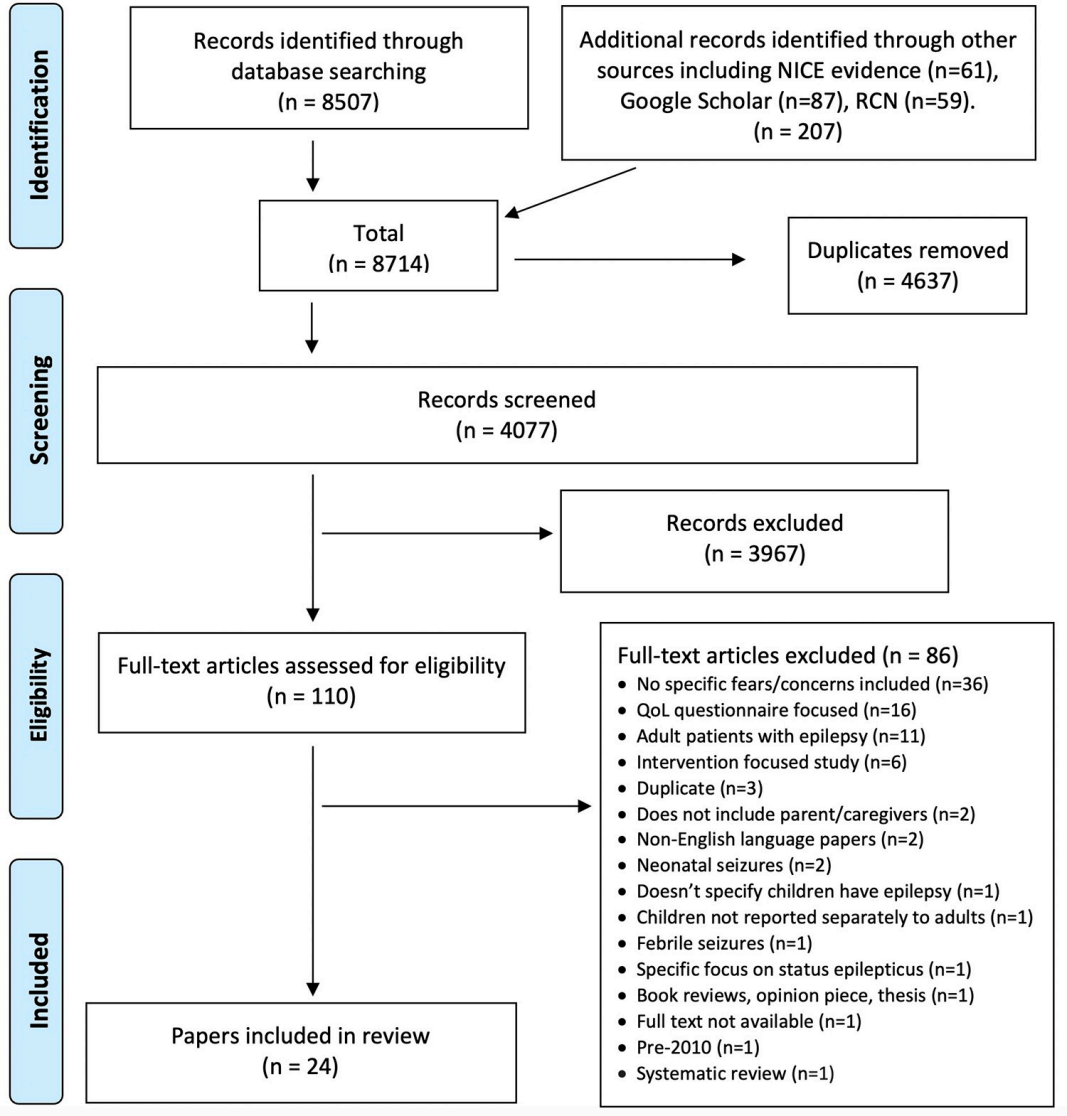
PRISMA flow chart of the scoping review search.

In Stage 1 papers (n = 8714) were imported into Covidence (systematic review software) for screening, duplicates (n = 4637) were removed. Titles and abstracts of all papers (n = 4077) were blind reviewed by at least two members of the review team (AR, GC, BC, AC and LB) and conflicts resolved by a third reviewer from the team. One paper was unavailable in full-text format and was excluded [31]. In Stage 2, members of the review team (AR, GC and BC), were allocated papers with two reviewers each screening all full-text papers (n = 110). Twenty-four papers met the inclusion criteria and aim of the review. Conflicts were resolved through discussion between the two reviewers or where further advice needed by a third reviewer (see Fig 1).

### Stage 4: Charting the data

We developed a data extraction sheet which we iteratively refined and included the following broad categories: fears and concerns, impact of fears and concerns, similarities/differences between children and parents/caregivers, and mothers and fathers, mitigations and risk factors. In line with the aims of a scoping review, all fears and concerns were included in the data extraction sheet (see S5 Table). Reviewers (BC, GC, AR, LB) independently extracted data from their allocated articles with extractions checked for consistency and condensed by one reviewer (BC).

### Stage 5: Collating, summarising, and reporting results

Information from the included papers was collated and summarised within the data extraction table (S5 Table) and this proved invaluable in helping to develop initial themes which were refined and finalised through discussions involving all members of the research team.

### Stage 6: Consultation

A key stage of Arksey and O’Malley’s [23] framework which is often not used within scoping reviews, is the sharing of review findings and consultation with members of the associated audience. We were determined to ensure our review modelled good practice and core to our review was engagement with parents in the Family Advisory Group who have experience of their children’s epilepsy at key stages during the review. This involved us asking for their input during one of their regular meetings about the scope of the review question, identification of search terms and key words and review of findings. So, for example, they were interested in our review encompassing both existing as well as future fears and concerns and we added this element to the review question. In reviewing our findings parents provided feedback on how the reviewed research resonated with their own thoughts and experiences.

## Results

The results section is structured to firstly present an overview of the characteristics of the included studies followed by the three themes: fears and concerns regarding their child’s epilepsy; impact of epilepsy-related fears and concerns on the daily lives of children and parents/caregivers; and impact of epilepsy-related fears and concerns on their social and emotional well-being.

### Overview of included studies

In total, 24 papers were included in the review and each paper was treated as a separate study; however, three papers by Webster [32–34] may be part of one study and two papers by Benson [35,36] may be part of one study.

Data are reported on studies undertaken in the USA (n = 4) [37–40], Ireland (n = 3) [35,36,41], Canada (n = 3) [42–44], UK (n = 4) [32–34,45] and one each in Australia [46], Brazil [47], Greece [48], India [49], Iran [50], Malaysia [51], Mali [52], Serbia [53], Sri Lanka [54], and Zimbabwe [55].

#### Appraisal of study quality

Whilst the appraisal of study quality is not an indicative element of Arksey and O’Malley’s [23] framework, the team felt it was important to consider quality as this would inform our reporting and discussion. The Mixed Methods Appraisal Tool (MMAT) v2018 [26] is an appraisal tool that accommodates appraisal of empirical studies using different methods; specifically it supports review of five categories of studies (qualitative, quantitative randomised controlled trials, quantitative non-randomised, quantitative descriptive and mixed methods). The MMAT was used to review, but not score, the methodological quality of the studies (S6 Table). In eight of the 24 studies methodological quality was impaired by poor or incomplete reporting [32,33,36,38,40,47,49,52]. The other key limitations to study quality were difficulty determining adequacy of method [38,47] or measurements [52] to address the research question, representativeness of sample [37,52], risk of non-response bias [37,52,53] and issues of integration of data within the mixed-methods studies[36,49].

#### Designs and methods

The studies adopted different designs and methods. Most (n = 19) used a qualitative design; of these, nine used a non-specific qualitative approach [32–34,38,40,41,45,51,54], three used a phenomenologically informed design [44,48,50], three used a descriptive design [43,47,55] and the other qualitative designs were exploratory (n = 1) [35], Grounded Theory (n = 1) [39], narrative (n = 1) [42] and realist (n = 1) [46]. Three studies used quantitative descriptive approaches [37,52,53] and two used mixed methods [36,49].

The most frequently used qualitative method (n = 20) was interviews. Of these, most (n = 9) were described as semi-structured [32–36,44,46,47,49], other interview approaches were in-depth (n = 5) [44,48,50,51,54], non-specific (n = 6) [38,39,41,43,45,55] and life narrative (n = 1) [42]. Six studies used more than one method [32,33,36,38,49,54]. One interview-based study also used auto-driven photo-elicitation interviews [33]; this is a method where participants take photographs that illustrate an aspect of their experience that can help trigger a deeper and more meaningful dialogue within the interviews [56]. Other qualitative approaches were focus groups in four studies [32,38,43,54] and group interviews were used in two studies [32,40]. One study used an expert panel [38]. Surveys or questionnaires were used in five studies [36,37,49,52,53] and one study used a clinical profile and scales [49].

In terms of qualitative analysis, most studies (n = 11) used a form of thematic analysis [32–36,40–42,45,46,50]. Three studies used content analysis [43,47,54]. Three studies used a phenomenological approach [44,48,51], four studies used Grounded Theory [32–34,39]. Two qualitative [38,55] and one mixed method study [49] did not report how they had analysed their qualitative data. Six studies handling quantitative data used both descriptive and inferential statistics [37,48–50,52,53], and one qualitative study reported using descriptive statistics [55].

#### Target population/participants and recruitment

All studies recruited parents/caregivers as participants. Two studies included other family members [42], including grandparents [54]. Children or youth participated in four studies [32,33,36,42]. Two studies included other key informants as participants, including paediatric neurologists [38], school teachers and public health staff [54].

Although some studies recruited through more than one route, the main recruitment approach (n = 17) was via hospital settings [35–38,40–43,47–55]. The other main route for recruitment for studies (n = 13) was via community support groups and/or charitable [32–36,38,40–43,47,49,55]. Two studies recruited via either a specialist registry/database [43,46], two via a previous study [39,42], one via postcodes in a large geographical setting [45] and one study did not clearly report recruitment setting [44].

#### Characteristics of parent/caregiver participants; number participating, mother/fathers

The number of parent/caregivers of children with epilepsy in each study ranged from 2–360 participants [42,52] with a total of 1936 participants (parents/caregivers, foster parents, grandparents) recruited across all studies of whom there were reported as mothers (n = 1315) and fathers (n = 384). Across the different study designs the number of parents/caregivers ranged widely: qualitative (n = 7–91) [44,47,48], quantitative descriptive (n = 213–720) [52,53] and mixed methods (n = 60–72) [36,49]. Although one study did not report gender of parent/caregivers [44], most studies recruited more mothers than fathers with only one study [46] reporting that no fathers were recruited.

#### Characteristics of the parents’/caregivers’ children who had epilepsy

The age range of parents’/caregivers’ children ranged from less than one year [37] to ≤ 18 years. Ten studies recruited children aged 5 or older [32–36,41,44,47,48,54]. Three studies did not report the age of the children [43,46,55], although studies were not included if participants were >18 years.

Eight studies did not report the gender of the child [38,40,41,43,44,47,52,55]. Although 15 studies reported gender [32–36,39,42,45,46,48–51,53,54], in five studies it is not clear if there is some double counting of child participants [32–36]. Of the remaining 10 studies [39,42,45,46,48–51,53,54], there was a reasonable balance between female (n = 265) and male (n = 246) participants.

#### Diagnosis, seizure type, frequency and duration of seizures of the parents’/caregivers’ children

All studies reported that the diagnosis of the parents’/caregivers’ children was epilepsy; one study also reported SUDEP [43], another reported the diagnosis as being syndromes such as Dravet syndrome [38] and two studies reported types of epilepsy (e.g. intractable epilepsy [40] or idiopathic generalized epilepsy) [51].

Eleven studies did not report the type of seizure [32–34,37,43,47,48,50,52,53,55]. The remaining studies reported seizure type although different reporting systems were used. For simplicity of reporting, seven main categories are presented tonic/clonic/generalised (n = 10) [35,36,39,41–43,45,46,51,54], absence (n = 8) [35,36,41,42,44,46,51,54], focal (n = 3) [39,45,51], intractable (n = 3) [35,38,40], complex partial (n = 2) [35,36], and syndrome related (n = 1) [38,40].

Most studies (n = 16) did not report the frequency of seizures [32–34,36–38,42–44,46–48,50–52,55]. When studies did report seizure frequency measurement occurred over different time periods. For simplicity of reporting the following categories are presented daily (n = 2) [35,41] although children in the study reporting on complex syndromes may have been having daily or frequent seizures as the mean number of seizures over the past month was reported as being 423 (SD 971), median 25 (range 0–3000). The other frequencies reported included about once per week [35,41], several times per week/more than once per week [35,54], about once a month [35,41], about monthly or more often [45], about yearly [35,41]. Some studies reported that some or all the children were either seizure-free or well-controlled (although definitions were either not reported or inconsistent) [35,39,41,49,53].

Some studies (n = 8) indicated the time since diagnosis [35,36,38,39,45,46,50,51], others (n = 7) reported age at diagnosis [35–38,41,46,48] and others (n = 5) reported age at seizure onset [42,44,45,53,54]. Four studies reported both age of diagnosis and time since diagnosis [35,36,38,46] and one study reported both time since diagnosis and age of seizure onset [45]. One study uniquely reported time from seizure onset to diagnosis [55].

### Parents’/caregivers’ fears and concerns regarding their child’s epilepsy

#### Parents’/caregivers’ reported fears associated with their child’s seizures

Eight studies reported specifically on parents/caregivers’ fears about their child experiencing seizures or concerns about the associated impact of these seizures [34,37,38,42,43,46,50,54]. Specifically, parents/caregivers feared overnight seizures [42], the unexpectedness of seizures [54], and seizures away from home [50]. Parents/caregivers also feared the potential implications on their child’s other body systems (e.g., brain [37]). Parents/caregivers were concerned about their child continuing to have seizures [46,54] and seizure activity worsening [37,45]. Seven studies reported specifically on parents’/caregivers’ fears of their child’s death [32,34,37,38,43,49,51] and two on serious injury [32,55] from seizures. Concerns about Sudden Unexpected Death in Epilepsy (SUDEP) were particularly emphasised [32,34,38,43] and, in some cases, this led to parents/caregivers experiencing sleep deprivation [38]. Parents’/caregivers’ fear of their child’s death during their child’s first seizure was also highlighted [51]. Where considered, there were no similarities or differences in the fears and concerns expressed by mothers and fathers [36,42,50] or differences between the knowledge or attitudes towards epilepsy expressed by mother and fathers [53].

Six studies reported on parents’/caregivers’ fears about their child being different or being treated differently [35,36,44,48,51,54]. Parents/caregivers feared their child being bullied, for example at school [36] and/or their child’s siblings [48]. Parents/caregivers also feared their child being physically different or having learning difficulties as a result of their epilepsy [54]. In one study non-disclosure was used to protect their child from any physical or emotional hurt [55].

#### Parents’/caregivers’ fears associated with uncertainty about their child’s epilepsy and concerns about their child’s future

Five studies specifically reported on fears of uncertainty [34,38,40,44,51]. Parents’/caregivers’ fears about the unpredictability of epilepsy symptoms were particularly noted [34, 40,44,51]. Parents/caregivers had concerns about the prognosis for their child [44] and concerns about not understanding or being adequately informed about their child’s condition [34]. One study explicitly reported on parental fears around communication; this involved fear of misinforming their child when communicating with them about their epilepsy and concerns about where to source appropriate information about their child’s condition [41].

Thirteen studies reported specifically on parental concerns about their child’s future [34,35,37–40,45–47,51,53–55]; with one study talking of how the epilepsy diagnosis had dashed their pre-conceived hopes and expectations for their child’s future [35]. Parents/caregivers were concerned about various aspects of their child’s future including development [45,53,55], health [38,51], future seizures [46,54] and medication use [46]. Specific concerns were reported about their child passing through key transitions such as school [39,47,51], puberty [34], independence [47,55], the possibility for future independent living [34,40,45,51,55], job prospects/ employment [34,51,54] and other tasks such as learning to drive [34]. In one study, where the impact of these fears about the future were considered, parents/caregivers were aware these could be demoralizing and counter-productive [46].

#### Parents’/caregivers’ fears about their child’s medication

Four studies specifically reported on parents’ concerns around medication [33,45,49,54]. Parents/caregivers feared adverse side effects of medications [33,37,49,54], with some feeling they lacked information about possible impact on their child’s learning and behaviour [45] and mental illness (term as used in the study) [33]. Some parents/caregivers reported desiring medication changes if side effects were deemed too great [33]. One study reported that parents/caregivers feared retribution from spirits for using modern medicine or a worsening of their child’s condition for administering medication [52].

#### Parents’/caregivers’ fears about their child’s well-being and mental health

Four studies reported on parents’/caregivers’ concerns for their child’s well-being or mental health [39,41,49,54]. Specifically, parents/caregivers had concerns about their child experiencing depression or low self-esteem [39] or negative comments upsetting their child and making them feel sad or stressed [54]. Some parents/caregivers were worried their child’s feelings could result in them developing a negative self-image [54]. Some parents/caregivers felt children with epilepsy required more social and practical support for psychosocial development [49]. Parents/caregivers feared epilepsy-related communication could result in a child feeling ‘singled out’ in comparison to their siblings, parents/caregivers were keen to avoid their child feeling different [41].

### Impact of epilepsy-related fears and concerns on the daily lives of children and parents/caregivers

#### Impact of parents’/caregivers’ fears on their parental roles, relationships and their family

Eight studies reported fears related to parental/caregiving roles [33,40,43,44,46,47,49,51]. Parents/caregivers struggled to trust anyone else to care for their child, including when their child was at school [44,49]. Six studies reported on the impact of epilepsy on parental behaviours; parents/caregivers assumed overprotective behaviours, were more vigilant or continuously watched out for any possible triggers of a seizure [33,40,43,46,47,51]. Epilepsy-related fears impacted relationships [38,42,55], adding complexity to romantic relationships [42], impacting marriage [55], and could leave broader relationships with friends and extended family strained [38,55].

Four studies reported on the impact of parents’/caregivers’ fears of epilepsy on the family [35,41,45,55]. Parents’/caregivers’ fears impacted family activities [45] and childcare options [35]. One study reported that parents/caregivers changed how they treated their child to avoid making their child with epilepsy feel ‘different’ [41].

One study reported the majority of parents/caregivers allowed their child to sleep in the bed with them as a coping strategy to mitigate fears over night time seizures [43]. Parents/caregivers reported a range of ways in which they attempted to mitigate their fears and concerns; these included striving for normalcy [35,46], comparing their child’s epilepsy diagnosis with more aggressive or disabling conditions [46], trying to maintain an optimistic outlook [46], meeting and speaking with other parents/caregivers of children with epilepsy [46,55] and engaging with religious or spiritual healing [49,54,55].

#### Impact of parents’/caregivers’ fears on their child’s daily lives and activities

Fourteen of the studies reported parental/caregiving fears and concerns for their child’s daily life and the activities they engage with [32,34–36,40,43–45,47–49,51,54,55]. Some of these fears related to parental/caregiving concerns of their child being away from them, for example on school trips [38,53,54]. Parents/caregivers were concerned about their child having a seizure when alone [54] or that there would not be adequate care available to look after their child a seizure occurred [38,51]. Specifically, they were fearful of their child’s participation in social activities because they were concerned about the risks involved in the activities [32] or because of stigma [36,51]. Some parents/caregivers restricted, limited or prevented the activities children could participate in [44,49,54] including sleepovers and travel [51] or sporting activities [35,36,44,49,50,54,55], and some mitigated risks by selectively disclosing the epilepsy condition on a ‘need to know’ basis [35,36].

Parents/caregivers were concerned that their child’s epilepsy could have a negative impact on their child’s invitations to and/or attendance at events such as playdates, parties and sleepovers [35,50]. They were also concerned about their child’s epilepsy resulting in their child experiencing social isolation [44,55] and negatively affecting their child’s friendships [35].

Where considered, there were negative emotional and social outcomes for parents/caregivers as a result of an inability for their child to attend events with feelings of disappointment and dissociation in family relationships, which perpetuated isolation [50].

#### Impact of parents’/caregivers’ concerns related to children’s education and learning

Seven studies reported the impact of parents’/caregivers’ epilepsy related fears on a child’s education or learning [34,39,44,48,49,54,55]. Despite their epilepsy-related fears, parents/caregivers encouraged their child to continue schooling [49]; one child was reported to be home schooled because of epilepsy-related concerns [44]. It was reported how due to parental/caregiving concerns some children missed school because of medication errors although the source of/reason for these errors was not clear [44], and one child was home schooled [44]. Parents/caregivers feared the impact of epilepsy on their child’s current and future education or learning, for example, at high school [39].

#### Parents’/caregivers’ fears related to the impact of their child’s epilepsy on their work and finances

Five studies reported fears related to the impact of their child’s epilepsy on their work and finances [38,42,50,51,55]. Three studies reported on the impact of the condition on work and some parents/caregivers quit their jobs because of their epilepsy-related fears and concerns or to help manage their child’s condition [42,50,51]. Some families structured their schedules to limit impacts on their jobs, whereas others had major issues with unemployment [38]. The impact of the condition on family finances was reported and the cost of medication was highlighted [38,55].

### Impact of epilepsy-related fears and concerns on parents’/caregivers’ social and emotional well-being

Emotional impact of parental fears and concerns. Five studies reported on the emotional impact of their child’s epilepsy with parents’/caregivers’ experiencing worry, shock, upset and anxiety [35,43,44,46,51]. Parents/caregivers feared the distressing aspects of witnessing the physical manifestations of seizures [35].

There were specific time points when or areas of concern where emotional reactions were experienced. This included shock, worry and upset during the child’s first seizure [51], devastation, confusion, and worry at the point of diagnosis [35,44], anxiety around the risk of SUDEP, which reduced when the low risk of SUDEP was explained [43], as well as feeling vulnerable and helpless if they could not control their child’s seizures [46]. Parents/caregivers also felt anxious and stressed about explaining their child’s condition to their child’s school community resulting in lacking a sense of belonging with the school [44], anger and sadness in response to the offensive reactions of others [35] and experienced negative emotions often motivated by negative stereotypes of epilepsy [47]. In one study, mothers blamed themselves or some were blamed by their in-laws for ‘causing’ the child’s epilepsy [51] and emotional impact was evident in one study where epilepsy was believed to be caused by bad spirits [51]. Parents/caregivers reported the impact of epilepsy related fears and anxiety on their sleep because of the need for increased vigilance [47] resulting in them feeling exhausted [38].

In one study where mothers and fathers’ emotional experiences at the point of diagnosis were investigated showed that mothers often experienced mental health issues while fathers experienced feelings of worry and anger (at healthcare team) as well as concern for their family [43]. Fathers also reported feeing uncertain and frustrated at the lack of practical approaches to managing SUDEP [43]. In another study, fathers feared the impact of their child’s condition on their own identity such as being viewed the father of a handicapped child [48].

### Study limitations

It is important to recognise the limitations reported by the authors of the reviewed studies. Commonly authors recognised sampling bias including non-probability sampling [55], self-selection bias [35]; 10 studies (encompassing both qualitative and quantitative methods) indicated that their samples may not be representative [38,41,43,45,47,49,51–53,55]. Furthermore, six studies recognised the limitations of small sample sizes which may not be representative all parents/caregivers of children and adolescents with epilepsy [39,40,42,47,49,54]. Five studies acknowledged bias due to the over-representation of mothers or female caregivers [35,36,40,45,46]. Nine studies acknowledged limitations relating to the child’s diagnosis of epilepsy, for example, recruitment was limited to specific types of epilepsy [35,41], where children had co-morbidities [46,54] had seizures that were well-controlled [39,48,51], had severe epilepsy [40] or unknown seizure type [55]. Finally, authors acknowledged limitations regarding the methods employed, including interview methods [45] or lack of validation of survey methods [49].

### Study recommendations for practice

Overall, five studies recommended good quality tailored communication between healthcare professionals and parents/caregivers [36,37,41,47,48]; although the evidence-base was variable. Five studies recommended that (some) parents/caregivers would benefit from some form of tailored psychological support at various stages of the trajectory (diagnosis onwards) (e.g., counselling) to help them manage some of the fears (stigma, SUDEP) and challenges (e.g., disclosure) they faced [36,43,46,48,50]. One study recommended that interventions should be developed to support parents/caregivers and/or promote resilience [40] and three specifically suggested that parents/caregivers could benefit from signposting to [37] or engagement with other parents/caregivers of children with epilepsy and with epilepsy-focused support groups [46,48]. Raising the awareness of healthcare professionals about the impact of epilepsy on the psychosocial health of parents/caregivers was recommended in one study [55]. Two studies identified the need for the development of good education resources to promote informed parents/caregivers and children [41,48]. Five studies recommended greater efforts should be made in terms of advocacy and improving public (e.g., schools, community) awareness and information about epilepsy as this could reduce issues such as stigma [36,49,50,52,53]. Three studies recommended that account should be taken of cultural sensitivities related to epilepsy [49,52,55]. Nine studies did not report recommendations [32–34,38,39,42,44,45,51].

### Study recommendations for future research

A variety of future research studies were proposed; these could be broadly categorised as child/parent focused, communication focused, and impact focused. Two studies proposed work addressing the resonance or differences in the perspectives of parents/caregivers and children in terms of concerns and/or knowledge [33,54]. Future communication-related research included work addressing better communication between parents/caregivers and healthcare professionals [45], supporting parents’ communication with their children [32], and considering a range of issues related to disclosure [32,35]. Two studies proposed future research should address impact; one wanted to develop a tool for assessing caregiver impact [38] and the other proposed to explore the best way of supporting parents/caregivers to navigate systems and access resources [45]. Eleven studies did not report any plans for future research [34,36,37,39,40,46,48,50–53].

## Discussion

This scoping review mapped key concepts from research related to parents’/caregivers’ fears and concerns regarding their child’s epilepsy, the impact of these fears and concerns on the daily lives of children and their parents as well as the impact of these fears on parental social and emotional well-being. It was evident from the findings that parents’/caregivers’ fears and concerns stemmed from more than their child’s seizures, and these had far-reaching influences on their parenting, the lifestyle and activities of their child and their family. What was less evident was parents thought would support them in allaying their fears and concerns.

In considering how to structure the discussion we were drawn to the model underpinning compassion-focused therapy (CFT) [57,58] as a means of explaining and exploring our findings. CFT has been used in many health contexts such as supporting parents of adolescents with mental health problems [59], grief therapy [60] and mediating trauma experienced by people with intellectual disabilities [61]. However, although we could not find CFT research related to epilepsy, there is work with adults with epilepsy and the use of self-compassion [62,63]. The CFT model provides a framework to understand why people may hold fears and concerns, the impact of these feelings and how people can manage their emotions. We saw resonance between the three affect systems underpinning CFT with our findings: threat (parents’/caregivers’ fears and concerns), drive (the motivation and measures parents/caregivers take to mitigate the threats) and soothe (the desire for parents/caregivers to experience a sense of security in relation to their child’s epilepsy). Within our discussion we draw on the concepts of threat and drive to explore parents’/caregivers’ ongoing fears and concerns and how they encompass ‘more than just seizures’ and that these have wide-ranging impact (affect) on children, parents/caregivers and their everyday life. We use the threat-drive-soothe principles from the CFT model as a framework for our discussion and as the basis for proposing that parents/caregivers may benefit when clinicians adopt a more compassionate understanding and acknowledgments of parents’/caregivers’ fears and concerns.

The fears identified in this review suggest that for parents/caregivers the traditional consideration and treatment agenda for children with epilepsy needs to extend beyond ‘just seizures’ (or seizure reduction with limited side effects). This recognition of the importance of changing the agenda around the traditional treatment of children with epilepsy is increasingly being called for both within the literature [64] and within charity led, online campaigns (e.g., ‘#EpilepsyIsMoreThanSeizures’) [65]. Indeed, this review was undertaken as part of a wider programme of research which is underpinned by a desire to change agendas in childhood epilepsy [66].

### Fears and concerns create a sense of threat

In the current review, it was clear that parents/caregivers experienced a wide range of threats in the form of fears, concerns and worries. The threat from these fears and concerns triggered a legitimate need to protect their child, but as noted in the CFT model [57,58] an over-active sense of threat can generate a negative emotional response. The findings show there was a pervasive fear associated with the unexpected and uncertain nature of parenting a child with epilepsy [34,38,40,44,51]. The review findings align with previous research that suggests that the challenge of managing the day-to-day care of their child can be exacerbated by the often unpredictable nature of their child’s condition [67].

The review findings reveal that the day-to-day reality of living with these fears resulted in increased vigilance [47], sleep deprivation [38] and negative emotional wellbeing, all of which may function to exacerbate parental fears, stress or result in overly restrictive or perceived protective behaviours [33,40,43,46,47,51]. Evidence from studies in the current review concur with previous research that parents of children with epilepsy and other long-term conditions experience increased negative emotional outcomes such as parenting stress [12,19,21,55,68] and perceived overprotective behaviours [69].

Evidence from studies in the current review suggest some of the parents’/caregivers’ fears reflected concurrent threats to their child’s existing circumstances such as their child’s seizures, the impact of these on the child and the impact of anti-seizure medication. Broader threats related to the societal implications such as social stigma [35–37,40,44,47,48,51,54,55] and/or disclosing [35,36,44,49–51] their child’s condition, as also seen in studies of children with long-term conditions [70–72]. However, other fears were more future-focused, for example pervasive fears about their child’s future health [37,38,51], opportunities and abilities [34,40,51,54,55]. These fears appear to reflect empirical evidence that children with epilepsy can have reduced medical, psychological, social and cognitive outcomes [73–75].

A key distinction emerged from studies in the current review between the threats, fears and concerns that parents had some control over (e.g., adopting protective behaviours such as limiting travel away from home, restricting child’s activity participation [44,49,50,54,55]) and ones where they had much less control (e.g., the child’s seizures [35,38,42,43,46,50,54] and the social implications of their child’s condition, such as bullying [36] and/or being treated differently [35,36,44,48,51,54]). Epilepsy-related fears about factors outside of parents/caregivers control commonly negatively impacted parents’/caregivers’ emotional and social well-being, as also seen in other studies of parents/caregivers of children with long-term conditions [19–21,76,77].

### The drive to protect their child from threat

As seen in the review findings, parents/caregivers were clearly motivated (driven) to protect their child from the various threats that they perceived, and whilst ‘drive’ can be positive an over-active sense of drive can generate a negative emotional response [57]. Evidence from the review highlights that parents/caregivers felt they lacked appropriate knowledge or information about their child’s condition [34] as well as awareness of where to source information about their child’s condition [41]. Potentially, better access to information and resources could positively influence both the threat and drive systems; certainly recommendations from the reviewed studies propose that parents would benefit from tailored information [36,37,41,47,48], professional support [36,40,46,48,50] and peer support [37,46,48]. For example, the current review findings highlight that perceived threats from disclosure resulting in selective disclosure, which echoes concerns held by children with epilepsy reported in previous work [6].

### The need for information and support; the search for security and regulation

As discussed above parents’/caregivers’ fears and concerns created a threat and a drive to try and protect their child. However, the findings of the review indicate that many of these fears continued to dominate their lives as little acknowledgments and/or support was available to mitigate these concerns. In the CFT model, the ‘soothe’ system aims to help regulate the threat and drive systems and when this does not happen people feel unsafe and insecure [57]. Parents/caregivers desired information, advice and support [33,41,45] in their search for a greater sense of security about their child’s seizures and those aspects of their child’s epilepsy that were ‘more than just seizures’. Where explanations are provided, for example about the low risk of SUDEP, anxiety can be reduced about this fear [43]. The recommendations of the studies in this review highlighted the current lack of and the need to offer psychological support to parents/caregivers to help them manage fears and the impact of these fears (threat).

The fears and concerns perceived by parents/caregivers identified in the current review represented a broad and significant threat. What is not clear from the review is to what extent the ‘threat agenda’ of parental/caregiving fears aligns with the often more seizure-focused ‘clinical agenda’ of clinicians and how this may impact meaningful dialogue within clinical consultations. The review findings align with other work that suggest that parents/caregivers and clinicians can have different perceptions about child seizures and their impact [78] and both the level and sources of threat. Such different, albeit overlapping, agendas can result in parents/caregivers not receiving answers to questions they may find difficult to frame and clinicians missing opportunities to provide information about issues they do not appreciate are of concern to parents/caregivers. As seen in other studies, clinicians may offer reassurance to parents that does not meet their needs and lacks meaning [79]. The review highlighted that many parents/caregivers may benefit from psychological support to help them manage fears and the impact of these fears (threat).

The review findings suggest that a collaborative ‘agenda’ for thinking about childhood epilepsy is necessary and could help frame clinical consultations in a way that could address the medical concerns of clinicians as well as mitigate the wider emotional, psychosocial and societal concerns of the parents/caregivers. Such an agenda could be developed between children with epilepsy, their parents/caregivers and clinicians and could build on existing work such as the core outcome set (COS) for childhood epilepsy [17] developed at an earlier stage of the CASTLE programme [66].

In the UK and elsewhere some, but not all children and parents/caregivers, will have access to an Epilepsy Specialist Nurse (ESN) whose remit is to provide parents with information, advice and support about their child’s medical condition but also on coping with the wider challenges [80]. Consistent support for all parents of children with epilepsy from an ESN and acknowledgments of their fears and concerns could be an important way of validating and mitigating the threat reported by parents/caregivers in the review. The potential for peer-to-peer parent support was suggested in the review [46,48] and has been shown to be beneficial in other studies of parents/caregivers caring for children with long-term conditions [81]. Benefit may result from structured group-based intervention to support the health and well-being of parents/caregivers, as already established for parents/caregivers of disabled children [82,83].

The discussion of the findings of the review within the three affect systems of the CFT model [57,58] has the potential to help HCPs/clinicians understand, acknowledge and actively address and validate the fears and concerns of parents/caregivers of children with epilepsy and could help frame clinical consultations to address parental/caregiving fears and concerns. The provision of appropriate information, knowledge, meaningful clinical reassurance [79] and peer support may contribute to parents experiencing a greater sense of security (soothe) about managing daily life and the challenges of caring for their child with epilepsy.

### Strengths and limitations

Although, overall, the studies were robust (e.g., had methodological coherence, appropriate research designs), the findings of this review are limited by poor or incomplete reporting in the studies, including incomplete reporting of type of epilepsy or type of seizure and/or severity of seizure and the child’s gender not always being reported. The transferability of the review findings is limited as most of the studies are based in Western/ developed countries and most participants were recruited via hospitals. This may be further exacerbated by the decision to exclude any non-English language studies. Another limitation is that although studies talk of parents/caregivers, there is a preponderance of mothers and typically parents/caregivers are considered separately. Although this could be considered a strength, the children with epilepsy represent a wide age range and a wide range of time since diagnosis. We did not include theses within the review which may be considered a limitation.

A strength of our scoping review is the engagement with parent/caregivers to frame our review question and inform subsequent thinking; this stage is often not undertaken but provided a meaningful contribution to the shaping of the work [84].

A further strength of the study lies in our engagement with parents who noted that our findings align with their lived experiences with one parent saying “I feel the conclusion [of the review] is spot on and awareness of the condition, as a whole, is massively lacking. This is something we as a family feel strongly about and it is vital that awareness is raised”.

### Implications for research

Future research should actively consider the views of fathers and/or both parents/caregivers. There is also a need for future research to explore how knowledgeable, prepared and confident HCPs/clinicians (epilepsy nurse specialists/consultants etc) and other parents/caregivers are in supporting parents around many of these key issues. The CASTLE programme has recognised the need to explore parent/caregiver, child and clinician perspectives and the next stage is to explore clinicians’ perspectives. Additionally, future work could involve a collaboration between parents/caregivers and HCPs to explore the issues/topics for inclusion on a compassionate and collaborative agenda around relevant fears/concerns.

### Implications for practice

The clinician priorities which can often shape the agendas for consultations may need to shift to encompass parents’/caregivers’ fears and concerns about things apart from actual seizures. Creating opportunities for parents to discuss issues with knowledgeable specialist epilepsy nurses and other HCPs and/or with other parents/caregivers could potentially mitigate factors that impact on parental/familial well-being and the child’s life and activities. Understanding the full range of parental fears and concerns about their child’s epilepsy may help HCPs to ensure they address the issues and/or signpost parents to relevant advice and support. These implications for practice would benefit from further research taking account of parents’ priorities and concerns.

## Conclusion

The fears and concerns experienced by parents/caregivers of children with epilepsy reach beyond their child’s seizures and medication and encompass short and longer-term psychosocial and societal threats that can mean that their child may be subject to stigma, bullying and being treated differently. Parents/caregivers manage their fears and concerns and adapt their parenting/caregiving to help keep their child safe. A collaborative ‘agenda’ that encompasses both the important clinical management issues (seizures and medication) and acknowledges and addresses parents’/caregivers’ fears and concerns could provide a new way of thinking about childhood epilepsy and mitigate the emotional, psychosocial, and societal concerns of the parents/caregivers.

## Supporting information

S1 Table Preferred reporting items for systematic reviews and meta-analyses extension for scoping reviews (PRISMA-ScR) checklist. (PDF)

S2 Table Population, concept and context framework: Key terms and words. (PDF)

S3 Table Medline search strategy. (PDF)

S4 Table Databases and sources searched. (PDF)

S5 Table Data extraction table. (PDF)

S6 Table Mixed Methods Appraisal Tool (MMAT). (PDF)
