# Supplementary material for: Parents’/caregivers’ fears and concerns about their child’s epilepsy: A scoping review
Source: PLoS One. 2022 Sep 6;17(9):e0274001. doi: 10.1371/journal.pone.0274001 (PMC9447888; doi:10.1371/journal.pone.0274001)
Supplement: S2 Table — (PDF) [file pone.0274001.s002.pdf]

**S2 Table: Population, Concept and Context framework: key terms and words**

| | Term | Keywords | MeSH |
|---|---|---|---|
| Population | Parent<br>Caregiver<br>Marriage | Parent<br>Famil*<br>Marri*<br>Caregiver*<br>Children with epilepsy<br>Mother*<br>Mum<br>Carer*<br>Father*<br>Sibling*<br>Brother<br>Sister | Parents+<br>Mothers+<br>Fathers+ |
| Concept | Fears | Fear*<br>Concern*<br>Worr*<br>Anxiet*<br>Experience*<br>Feeling*<br>Anxious*<br>Hypervigilance<br>Living on the edge<br>Reassurance<br>Assurances | Fear+<br>Anxiety+<br>Psychological Distress+ |
| | Paediatric epilepsy.<br>Childhood epilepsy.<br>Seizure. | Paediatric epilepsy.<br>Rolandic epilepsy.<br>Benign Rolandic epilepsy.<br>Childhood epilepsy.<br>Seizure.<br>Sudden Unexpected Death in Epilepsy.<br>Suffocation. | Epilepsy+ |
| Context | All settings considered | | |
