# Supplementary material for: Parents’/caregivers’ fears and concerns about their child’s epilepsy: A scoping review
Source: PLoS One. 2022 Sep 6;17(9):e0274001. doi: 10.1371/journal.pone.0274001 (PMC9447888; doi:10.1371/journal.pone.0274001)
Supplement: S3 Table — (PDF) [file pone.0274001.s003.pdf]

S3 Table: Medline search strategy

The search strategy was as follows:

- TI/AB (“fear*" OR "concern*" OR “worr*” OR “anxiet*” OR “experience*” OR “feel*” OR “anxious*” OR “hypervigilance” OR “Living on the edge” OR “Reassurance” OR “stress” OR “psych*”) OR MH (Anxiety+ OR Psychological Distress+ OR Fear+);
- TI/AB (“parent” OR “parents” OR “family” OR “families” OR “marri*” OR “caregiver*” OR “Children with epilepsy” OR “mother*” OR “mum*” OR “carer*” OR Sibling* OR “dad*” OR “father*” OR “brother*” OR Sister*”) OR MH (Fathers OR Mothers OR Parents);
- TI/AB (“epilep*” OR “Paediatric Epilepsy” OR “Rolandic Epilepsy” OR “Childhood epilepsy” OR “Benign Rolandic Epilepsy” OR “seizure*” OR “Sudden Unexpected Death in Epilepsy” OR “Suffocation”) OR MH (Epilepsy+).
