# Supplementary material for: Parents’/caregivers’ fears and concerns about their child’s epilepsy: A scoping review
Source: PLoS One. 2022 Sep 6;17(9):e0274001. doi: 10.1371/journal.pone.0274001 (PMC9447888; doi:10.1371/journal.pone.0274001)
Supplement: S4 Table — (PDF) [file pone.0274001.s004.pdf]

S4 Table: Databases and sources searched

Databases	Grey Literature	Other
Medline.<br>CINAHL.<br>PsychInfo.<br>Scopus.	Open Grey.<br>Google Scholar.<br>NICE Evidence.<br>Royal College of Paediatrics and Child Health.<br>Royal College of Nursing.<br>National Institute of Health Research portfolio.<br>Department of Health.<br>Epilepsy Action.<br>Epilepsy Society.<br>Epilepsy Research UK.<br>Young Epilepsy.<br>Epilepsy 12.<br>International Bureau for Epilepsy (IBE).<br>International League Against Epilepsy (ILAE).<br>Links to international organisations: https://www.epilepsy.org.uk/about/international-epilepsy-organisations<br>Relevant conferences.	Reference lists.<br>Hand searching of key journals:<br>• Seizure (https://www.seizure-journal.com);<br>• Epilepsy and Behaviour (https://www.journals.elsevier.com/epilepsy-and-behavior); and<br>• Epilepsia (https://onlinelibrary.wiley.com/journal/15281167).
