# Supplementary material for: Parents’/caregivers’ fears and concerns about their child’s epilepsy: A scoping review
Source: PLoS One. 2022 Sep 6;17(9):e0274001. doi: 10.1371/journal.pone.0274001 (PMC9447888; doi:10.1371/journal.pone.0274001)
Supplement: S5 Table — (PDF) [file pone.0274001.s005.pdf]

| Author name, Year, Country | Research aims and/or research question | Design; Methods; Participants | Participant recruitment; Eligibility | Parents: Gender/role, Age.<br>Children: Gender, Age | Children: Diagnosis/seizure type; Other | Data analysis approach | Fears and concerns regarding their child's epilepsy | Impact of epilepsy-related fears and concerns on the daily lives | Impact of epilepsy-related fears and concerns on social and emotional well-being | Similarities or differences in the fears and concerns expressed by mothers and fathers | Similarities and differences in fears and concerns from the perspectives of children and their parents | Any mitigating or risk factors for parents fears or concerns | Study limitations | Study recommendations | Future research |
|---|---|---|---|---|---|---|---|---|---|---|---|---|---|---|---|
| Amjad et al. 2016<br><br>Iran | To explore the experience of parents of children with epilepsy. | **Design:** Qualitative, phenomenological.<br><br>**Methods:** Prolonged, engaged, in-depth interviews.<br><br>**Participants:** Parents | **Recruitment:** Purposive sampling via 2 referral governmental hospitals in Tehran.<br><br>**Key eligibility:** Parents only one child with grand mal epilepsy aged 1-18 years with diagnosis of epilepsy made at least one year ago. | **Parents:** n=10<br>**Gender/role:** female (n=5),male (n=5).<br>**Age:** range 20-42yrs (mean 34.1yrs).<br><br>**Children:** n=10.<br>**Gender:** female (n=5),male (n=5).<br>**Age:** Not reported. | **Diagnosis:** Epilepsy.<br><br>**Time since diagnosis:** Children had been diagnosed for 1-11 yrs. | Thematic analysis. | **Fear of seizures:** parents avoid staying away from home, so they can feel prepared for an attack and they can react quickly.<br><br>**Fear of unavailability of basic health care** means travel avoided.<br><br>**Fear of crowds/parties:** as thought to increase the risk of seizures.<br><br>**Fear of disclosure:** avoid parties, ceremonies and social functions. | **Impact on work/finances:** Mostly mothers quit jobs because of their fears and concerns about their child's seizures and the consequences. | **Fear of seizure occurring in public:** inability to attend parties or events, which can lead to disappointment and dissociation in family relationships, followed by further isolation. | Not reported. | Not applicable. | Restriction of travel. | Not reported | Pay special attention to these families and provide help (e.g., nurses).<br><br>Provide parents with counselling and education and use mass media to increase public awareness about epilepsy is also necessary. | Not reported. |
| Benson et al. 2016<br><br>Ireland | To present the stigma experiences of CWE and their parents, in the context of communicating about epilepsy within and external to the family unit. | **Design:** Mixed methods (sequential exploratory).<br><br>**Methods:** Qualitative semi-structured interviews and a quantitative, cross-sectional survey.<br><br>**Participants:** Parents and CWE. | **Recruitment Phase 1:** Purposive sampling via a paediatric neurology unit and a national epilepsy association.<br><br>**Key eligibility:**<br>CWE: 6-16 years, prescription for antiepileptic drugs, no other significant medical condition and/ or developmental delay. Parent participants: parent/ primary caregiver of the participating child..<br><br>**Recruitment Phase 2:** CWE and/or parents recruited through paediatric/neurology units in regional/national hospitals or via national epilepsy association.<br><br>**Key eligibility:** CWE and parents were eligible to participate if they (or their child) had a diagnosis of epilepsy and were aged 8-18 years and no other significant medical condition and/or developmental delay. | **Phase 1:**<br>**Parents:** n=40<br>(34 families).<br>**Gender/role:** mothers (n=26), fathers (n=2), both parents (n=6).<br>**Age:** not reported.<br><br>**Children:** n=33.<br>**Gender:** female (n=20), male (n=13)<br>**Age:** range 6-16yrs (mean 11.14, SD 2.91).<br><br>**Phase 2:**<br>**Parents:** n=72.<br>**Gender/role:** female (n=66), male (n=6).<br>**Age:** range ≤25 yrs to ≥56 yrs; most (n=54, 75%) 41-55 yrs.<br><br>**Children:** n=47.<br>**Gender:** female (n=25), male (n=22).<br>**Age:** range 8-18yrs, (mean 13.19, SD 2.82). | **Diagnosis:** Epilepsy.<br><br>**Seizure type:** absence (n=30), tonic-clonic (n=22), complex partial (n=11), myoclonic (n=10), simple partial (n=8), clonic (n=5), tonic (n=4), atonic (n=2).<br><br>**Age of seizure onset:** range 0.58-16 yrs (mean 9.04, SD 3.64).<br><br>**Time since diagnosis:** range 0-12 yrs (mean 4.15, SD 2.95).<br><br>**Seizure frequency:** daily (n=3), several times a week (n=3), about once a week (n=3), about once a month, (n=2), less than monthly (n=15), about once a year (n=5), seizure-free (n=14), unknown (n=2). | **Qualitative:** Thematic analysis.<br><br>**Quantitative:** Descriptive and inferential correlational statistics. | **Fear of social exclusion and social stigma:** caused their child's social participation to be unnecessarily curtailed.<br><br>**Fear of child being bullied/teased:** peer bullying at school.<br><br>**Fear of disclosure:** disclosure on need to know basis. | **Child's social participation curtailed:** due to fear of stigma etc. | **Fear of child being bullied/teased:** peer bullying at school. | Not reported. | Stigma-related concerns similar to parents.<br><br>**Exclusion by peers from social activities:** sometimes caused by parental fear.<br><br>**Epilepsy-elated bullying:** by peer group.<br><br>**Concealment of epilepsy:** aimed to avoid epilepsy stigma. | Concealment and/or selective disclosure of epilepsy. | Data only gathered from CWE and one parent and not other family members.<br><br>Over-representation of mothers which may obscure gender perspective differences. | Communication-based interventions to encourage HCPs to actively engage with CWE / parents about their epilepsy-related stigma experiences and perceptions.<br><br>CWE/parents may require assistance in learning how to initiate, manage and maintain open conversations about epilepsy-related issues within the home environment and with disclosure. | Not reported. |
| Benson et al. 2017<br><br>Ireland | To explore the challenges parents of children with epilepsy (CWE) experienced when deciding to disclose their child's epilepsy diagnosis to others | **Design:** Qualitative exploratory.<br><br>**Methods:** exploratory semi-structured interviews.<br><br>**Participants:** Parents | **Recruitment:** Parents via a neurology clinic of a specialist children's hospital and from a national epilepsy association.<br><br>**Key eligibility:** Parent(s)/ guardian(s) of a child with a diagnosis of any type of epilepsy; a prescription for anti-epileptic drugs and (no significant intellectual disabilities, learning difficulties and/or developmental delay. | **Parents:** n= 34 (of 29 CWE).<br>**Gender/role:** mother (n=27); fathers (n=7).<br>**Age:** Not reported.<br><br>**Children:** n=29.<br>**Gender:** female (n=17), male (n=12).<br>**Age:** range 6-16yrs (mean 7.35, SD 2.85) | **Diagnosis:** Epilepsy.<br><br>**Seizure type:** multiple TYPES (n-=19), tonic-clonic (n=19), absence (n=14), one type only (n=10), complex partial (n=10), myoclonic (n=6), tonic (n=5), simple partial (n=4), atonic (n=4), electrical status epilepticus in sleep (ESES) (n=1).<br><br>**Age at diagnosis:** range 2-14.5yrs (mean 7.35 yrs, SD 3.20).<br><br>**Time since diagnosis:** range 0.17-10 yrs (mean 3.87, SD 2.87).<br><br>**Therapy:** Most children receiving polytherapy (n=16) or mono therapy (n=12).<br><br>**Seizure freedom:** Only 41.3% of parents reported child had been seizure free for 4 weeks at the time of interview | Thematic analysis | **Fear of disclosure:** some parents (especially of younger children) viewed concealment and/or selective disclosure management strategies as protective of their child's psychosocial wellbeing. Fear of disclosure elected parental emotions of concern, worry and upset.<br><br>**Fear of child being treated differently:** Parents strove to foster a sense of normality and support child to reach his/her 'potential'.<br><br>**Fear associated with seizures:** seizure manifestations deemed worse in comparison physical manifestations of many other more visible, chronic conditions. Physical manifestations can be intrusive, startling, fear-evoking and distressing to witness. | **Impact/fear on child's opportunities and participation being restricted:** stigmatisation, prejudiced attitudes, discrimination, and/or exclusion from social, recreational and/or sporting activities.<br><br>**Impact on family:** difficulty finding someone to care for the child in their absence. | **Impact of diagnosis:** period of grief as parents grappled with the loss of their 'healthy' child; feelings of 'devastation' 'upset', 'concern', 'worry' and 'shock'.<br><br>**Fear of being treated differently:** based on people knowing about child's diagnosis.<br><br>**Concerns about impact on socialisation:** fear of potential impact on invitations to playdates, parties and sleepovers, and impact on their child's friendships.<br><br>**Impact/fear of negative reactions to disclosure:** Fear of and actual negative responses ended to mean parents maintained secrecy about diagnosis or selectively disclose.<br><br>**Impact on parents:** offensive reactions, e.g., others mimicking seizures; this elicited negative emotions among parents (e.g., anger, concern, sadness, disappointment).<br><br>**Impact on future:** epilepsy diagnosis had dashed and/or altered parents' pre-conceived hopes and expectations for the future of their child (e.g., academic, romantic). | Not applicable. | Not applicable. | Concealment and/or selective disclosure of epilepsy.<br><br>Seeking normalcy. | Findings are limited to parents of children with intractable epilepsy and who experienced challenges with disclosure of their child's diagnosis.<br><br>Fathers were underrepresented in this self-selected parent population.<br><br>Self-selection bias more probable for sample recruited via the national epilepsy association.<br><br>Sample not heterogenous. | A facilitative environment needs to be created in which it is the norm rather than the exception for parents to openly discuss their child's epilepsy with others.<br><br>Advocacy efforts should be heightened, with HCPs, patient advocacy groups and healthcare organisations taking a more active role in striving to increase the visibility of epilepsy within the public domain and tackling misconceptions about epilepsy | Longitudinal design is required to elucidate whether the adoption of concealment and/or selective disclosure strategies is problematic or protective.<br><br>Identify the contexts in which parental epilepsy disclosure is most critical.<br><br>Investigate the bidirectional relationship between stigma and disclosure.<br><br>Explicate how epilepsy-related stigma (and specifically felt stigma) can best be eradicated. |
| Fayed et al. 2021<br><br>Canada | To identify themes from youth and family narratives to inform the process of optimizing a good life with active epilepsy. | **Design:** Qualitative narrative.<br><br>**Methods:** Life narrative interviews.<br><br>**Participants:** Youth and family members | **Recruitment:** Purposive sampling of youth from neurology ambulatory clinics at McMaster Children's Hospital and via QUALITE study. Additional sampling via epilepsy community groups.<br><br>An identified family member were also invited to participate.<br><br>**Key eligibility:** Youth participants had to be 8-30yrs and have active epilepsy. | **Family members:** n=8 (mother, father, sister).<br><br>**Youth:** n=7 *(although only 2 eligible for inclusion in this review).*<br>**Gender:** female (n=3), male (n=4) *(only 2 males eligible for inclusion in review)*<br>**Age:** range 18-30yrs *(but only the 18yr olds included in this review).* | **Diagnosis:** Epilepsy.<br><br>**Age at onset:** 7yrs, 13yrs *(for eligible children).*<br><br>**Seizure types:** absence (n=5), generalized tonic-clonic (n=6) *(Note: eligible for inclusion in this review, Absence, Generalised tonic-clonic (n=1)and. Gneralised tonic-clonic (n=1).* | Thematic analysis | **Fear associated with seizures:** Parenting overnight- fear of catastrophic seizure in sleep. | **Impact on work:** father giving up work (personal sacrifice) to help manage child's condition. | **Impact on parental well-being:** importance of support from wife to help 'being there' for child.<br><br>**Impact on relationships:** Epilepsy adds a layer of complexity to romantic relationships<br><br>**Impact on youth well-being:** worrying about impact of missing school and having to catch up.<br><br>**Impact/fear of negative reactions to disclosure:** youth's decision not to disclose due to fears of being thought different. | Not reported. | *Not reported specifically for the 2 youth included in review.* | *Not reported specifically for youth in this review.* | Small sample of 7 (of whom only 2 eligible for inclusion this review).<br><br>Method excluded youth who could not participate in interview process. | Not reported. | Use of methods that apply the life course-multiple perspectives approach to lower functioning groups. |
| Fowler et al. 2021<br><br>USA | To describe the knowledge and fears of parents of children diagnosed with epilepsy in the hospital and clinic settings. | **Design:** Descriptive, comparative.<br><br>**Methods:** Scale and questionnaire.<br><br>**Participants:** Parents | **Recruitment:** Convenience sample via paediatric neuro/ ortho hospital unit or paediatric neurology clinic.<br><br>**Key eligibility:** Parents of children diagnosed with epilepsy | **Parents:** n=40.<br>**Gender/role:** female 80%.<br>**Age:** range 21-69yrs (mean 39).<br><br>**Children:** n=40<br>**Gender:** female (n-16), male (n=24).<br>**Age:** <1yr-18yrs (mean 8.5yrs). | **Diagnosis:** Epilepsy.<br><br>**Age at diagnosis:** (51%) of parents reported that epilepsy was diagnosed prior to 'first grade' (most often at <1 year to 3 years). | Descriptive statistics for demographics and survey responses, and t-test for comparison between parent groups. | **Fear of seizures:** what to do if child has a seizure and fear their child might die during a seizure.<br><br>**Fear of impact of seizures:** will lead to brain damage.<br><br>**Fear of future seizures:** that the seizures will get worse and have them lifelong.<br><br>**Fear associated with medications:** that medication will be harmful in long-term.<br><br>**Fear of social stigma:** afraid of what could happen if people look at their child. | Not clearly reported. | Not reported. | Not reported. | Not reported. | Not clearly reported. | Not reported. | Talk to parents about their fears related to epilepsy and provide information to allay fears and signpost to support groups. | Not reported. |
| Gazibara et al.<br>2014<br>Serbia | To assess knowledge, attitudes, and behaviors of parents whose children were diagnosed with epilepsy | **Design:** Quantitative descriptive (cross-sectional).<br><br>**Methods:** Questionnaire.<br><br>**Participants:** Parents | **Recruitment:** Convenience sampling via an outpatient department of the Child and Adolescent Neurology and Psychiatry Clinic, Belgrade.<br><br>**Key eligibility:** Child diagnosed with epilepsy by a paediatric neurologist. | **Parents:** n=213.<br>**Gender/role:** mother (n=164); father (n=49).<br>**Age:** range 24-69 yrs (mean 43 ± 8.2 years).<br><br>**Children:** n=213<br>**Gender:** female (n=114), male (n=99)<br>**Age:** range ≤6-18yrs (mean 13.4 ± 6.1 yrs). | **Diagnosis:** Epilepsy.<br><br>**Age at seizure onset:** mean 6.4yrs.<br><br>**Epilepsy control:** 84.5% considered well controlled. | Descriptive and inferential statistics. | **Concerns about the child's future development:** associated with concerns about child's personal development.<br><br>**Concerns related to supervision of child away from home:** e.g. extended school trips.<br><br>**Concerns about social support:** more social support needed. | Nothing specific reported here. | Nothing specific reported here. | No statistical differences between the reports of mothers and fathers | Not applicable. | Stronger positive parental attitude towards childhood epilepsy was influenced by higher monthly income and having more children. | Parents who participated may not reflect those who declined. | Principal goal of health-care service should be ensuring education and support at the community and school levels (e.g., training programmes strategies to reduce anxiety and emotional strain) for both parents and children with epilepsy. | |
| Jensen et al. 2017<br>USA | To identify the most important domains that should be assessed in an efficient measure of caregiver impact for caregivers of children and young adults with severe epilepsy. | **Design:** Qualitative.<br><br>**Methods:** Expert panel, focus groups and telephone interviews.<br><br>**Participants:** Paediatric neurologists and caregivers. | **Recruitment:** Purposive recruitment of 8 paediatric neurologists, from USA, France, Belgium, UK; female (n=3),male (n=5), known to investigator or colleagues of investigators to expert panel. Recruitment of caregivers via Dravet Syndrome Foundation and epilepsy social media sites.<br><br>**Key eligibility:** Paediatric neurologists providing healthcare services to children and young adults with epilepsy. Caregivers providing care to children with Dravet syndrome or other severe epilepsy conditions(e.g., SCN8A epilepsy, Lennox-Gastaut syndrome, Doose syndrome) severe epilepsy. | **Primary caregivers:** n=19;<br>**Gender/role:** female (n=16), male (n=3); biological parent n=18.<br>**Age:** range 32.5-55.8 yrs (mean 42.0, SD 6.4 yrs).<br><br>**Children:** n=not reported<br>**Gender:** not reported<br>**Age:** range 1.7-17.8 yrs (mean 8.4, SD 5.2yrs). | **Diagnosis:** Dravet syndrome (n=12), SCN8A (n=3), Lennox-Gastaut syndrome (n=1), Doose syndrome (n=1), Intractable epilepsy (n=1), Epilepsy with developmental delay (n=1).<br><br>**Age at diagnosis:** mean 1.9 years (SD 5.2).<br><br>**Time since diagnosis:** mean 6.4 years (SD 4.4).<br><br>**Number of seizures, past month:** Mean 423 (SD 971); median 25 (range 0-3000). | Specific method not reported but data 'coded and analysed'. | **Fear is 24/7:** uncertainty during day and night about child's epilepsy; child's epilepsy redefines caregivers sense of purpose in life.<br><br>**Fear of SUDEP or seizures during sleep:** linked to caregiver sleep deprivation.<br><br>**Fear of leaving child:** other people may not be adequately trained or able to provide adequate care if a severe seizure or other health complication occurs.<br><br>**Fear for future:** Worries about child's health and future. | **Impact of sleep deprivation on caregivers:** lack of restful sleep due to fear of seizures/SUDEP.<br><br>**Impact of exhaustion:** lack of energy to meet own self-care needs (staying fit, eating healthy foods).<br><br>**Impact on finances:** financial impact often reported as greatest during the early stages of diagnosis and initial treatment. Some families experienced treatment-related costs.<br><br>**Impact on work:** Some families structured schedules so jobs not affected, others had major issues with employment. | **Impact on emotional well-being:** caregivers often experienced psychological distress due to demands of caregiving and experienced feelings of anger, anxiety, guilt, and helplessness.<br><br>**Impact on time for self:** caregivers found it difficult to find time for themselves.<br><br>**Impact on relationships:** focus on child often left spouse, friend, and extended family relationships strained. | Not reported. | Not applicable. | Not reported. | No participants from Asia, Africa, or South America so may not reflect caregiving impact domains from these areas/cultures.<br><br>All participants fluent English speakers, willing to find time to participate and from well-resourced countries. | Not reported | Develop a comprehensive or efficient measure for assessing caregiver impact among caregivers of children and young adults with severe epilepsy. |

| | | | | | | | | | | | | | | | |
|---|---|---|---|---|---|---|---|---|---|---|---|---|---|---|---|
| Jones et al.<br>2014<br>USA | To develop a theoretical framework to aide clinicians and researchers to more effectively work with parents to address the child's needs, utilizing a person-and family-centered care model. | **Design:** Qualitative, Grounded Theory.<br><br>**Methods:** Interview.<br><br>**Participants:** Parents. | **Recruitment:** Recruited via a pilot study; half were parents who participated and half were parents who did not participate in pilot study.<br><br>**Key eligibility:** Parents of children with epilepsy. | **Parents:** n=22.<br>**Gender/role:** mother (n=17); father (n=5).<br>**Age:** range 31-53yrs (mean 40.5, SD 6.4yrs).<br><br>**Children:** n=22<br>**Gender:** female (n=11), male (n=11)<br>**Age:** range 9-18yrs (mean 12.52, SD 2.5yrs). | **Diagnosis:** Epilepsy.<br><br>**Seizure type:** generalised (n=14), focal (n=8).<br><br>**Time since diagnosis:** 5.57 years (SD 3.36)<br><br>**Seizure-related medication:** one medication (64%), more than one medication (n=3), no medication (23%).<br><br>**Seizure control:** All children were currently well controlled with 1 to several seizures (less frequent than monthly) per year.<br><br>**Comorbidities:** included anxiety, depression, ADHD, autism, intellectual disability, and learning disability. | Grounded theory. | Not reported specifically but described in terms of struggles. | **Impact on education:** Concerns about education/managing at school and learning and also future supports in high school.<br><br>**Impact on parents' responses to child's struggles:** 3 zones (1) struggles not seen as problematic, active support not sought; (2) struggles seen as problematic, active support and interventions sought; (3) struggles seen as problematic, and intervention sought in more solution-focused manner. | **Impact on child's mental health:** concerns about depressive symptoms and their severity and cancer; concerns about low self-esteem<br><br>**Impact on education:** Concerns about education/managing at school and learning and also future supports in high school. | Not reported. | Not applicable. | Age of child, parent, and seizure onset, degree of seizure control, medication, parent education level, child's academic success, parent's marital status influenced which zone they were in. | Small study with small sample size. All children well controlled, most on one seizure medication, known to have co-occurring problems so findings not necessarily transferable. Unclear if the parents of children with no comorbidity would fit into the zone framework outlined. | Not reported. | Not reported. |
| Jones et al.<br>2019<br>UK | To gain a comprehensive understanding of the experiences and needs of parents of young children with 'active' epilepsy (on AEDs and/or experienced at least one seizure in the last year). | **Design:** Qualitative<br><br>**Methods:** Interviews.<br><br>**Participants:** Parents/guardians | **Recruitment:** Parents/guardians of eligible children were identified via a link paediatrician on the research team.<br><br>**Key eligibility:** Parents of children with epilepsy at least one year of age, with current 'active' epilepsy and resident in specified postcodes. | **Parents:** n=47<br>**Gender/role:** Mother (n=38), father (n=9).<br>**Age:** Not reported.<br><br>**Children:** n=40<br>**Gender:** female (n=17); male (n=23).<br>**Age:** range 1-7yrs (mean 4.60, SD 1.49 yrs). | **Diagnosis:** Epilepsy.<br><br>**Predominant seizure type:** generalized (n=20), focal (n=20).<br><br>**Duration of epilepsy:** range 0.28-6.52yrs (mean 2.97, SD 1.58yrs)<br><br>**Age of seizure onset:** range 0.04-6.00yrs (mean 1.65, SD 1.34 yrs).<br><br>**Seizure frequency:** monthly or more often (n=29), less often (n=11).<br><br>**Seizures longer than 30 min:** (n=7).<br><br>**Required rescue therapy:** (n=24).<br><br>**Developmental level:** >2SD below mean: (n=30). | Thematic analysis | **Fear of possible impact of AEDs:** fear of impact on child's behaviour and learning and inadequate information about possible impact of side effects.<br><br>**Future concerns for the child:** concerns especially related to children with significant developmental delay as likely to have difficulty living independent lives. | **Impact on family activities due to seizures or associated difficulties:** Family holidays, shopping and other activities often cancelled/rescheduled because of concerns about the child's epilepsy or associated difficulties on the activity. | **Impacts associated with child's developmental delay and challenging behaviour:** feelings of loss that the child may not achieve the same milestones as children without such problems or have the life they had anticipated. | Not reported | Not reported. | Not reported. | Majority of respondents were mothers. Interviews conducted either in person or telephone; mode may affect content and length of responses. Findings may not be relevant for children < 1yr or >7yrs. Participants all from one defined geographical region of UK. | Not reported. | How best to inform parents of the nature of both epilepsy and associated conditions as well as how best to support parents' informational and emotional needs after diagnosis.<br><br>Adapt psychoeducational interventions in childhood epilepsy for digital use and subsequently evaluate.<br><br>Investigate best ways to support parents to navigate the health and education system so as to access effective educational and therapeutic provision. |
| Kampra et al.<br>2017<br>Greece | To explore the challenges that Greek parents/caregivers of CwE face to provide useful knowledge for healthcare professionals about this population's needs. | **Design:** Qualitative, hermeneutic phenomenological (with statistical component).<br><br>**Methods:** In-depth interviews.<br><br>**Participants:** Parents. | **Recruitment:** Convenience sample parents/caregivers of children with epilepsy whose children were consecutive patients of the outpatient neurology clinics of two public hospitals in Athens.<br><br>**Key eligibility:** Parents of children with controlled epilepsy, no significant intellectual disabilities, learning difficulties, and/or developmental delay, and attending regular school. | **Parents:** n=91.<br>**Gender/role:** female (n=60); male (n=31);<br>**Age (female):** range 24-68yrs (mean 42yrs).<br>**Age (male): range 27-68yrs (mean 47yrs).**<br><br>**Children:** n=91.<br>**Gender:** female (n=60); male (n=31)<br>**Age:** not reported for sample although eligibility 5-17yrs. | **Diagnosis:** Epilepsy<br><br>**Age of diagnosis:** <5yrs (n=24), 5-9yrs (n=38), 10-15yrs (n=22), 16-17yrs (n=2), don't remember (n=5). | **Qualitative:** van Manen's process (phenomenological).<br><br>**Quantitative:** Descriptive and inferential statistics | No fears directly about epilepsy reported. | **Impact of bullying:** some parents hesitated to discuss epilepsy with their child because of the fear of bullying or isolation from his/her peers and inability of chid to cope with disclosure.<br><br>**Impact of social isolation:** some parents feared social exclusion of their child.<br><br>**Impact on social image:** some parents kept epilepsy secret as feared consequences on child's and family social image: ,<br><br>**Impact of stigma:** some parents didn't disclose to school if child seizure-free during day due to fear of bullying.<br><br>**Impact of inadequate information to support coping:** parents needed more information and support (e.g. from schools, drs) to help them cope). | **Impact on siblings:** concerns about siblings being bullied because of epilepsy.<br><br>**Impact on social image of their family:** concerns about bullying or isolation incurred by the stigma around epilepsy.<br><br>**Need for emotional and practical support:** gained emotional support by talking with close friends and relatives.<br><br>**Impact on child's social integration and well-being:** concerns about how other people would see child and treat them.<br><br>**Impact on parental identity:** being seen as father of handicapped child.<br><br>**Impact of negative aspect higher in small rural communities:** parents from small communities more worried about disclosure. | Not reported. | Not reported. | **Level of parent education:**(tertiary educated parents talked to child about epilepsy at a higher rate than primary/secondary educated parents) ($p = 0.013$).<br><br>**Parents' employment condition:** employed parents talked with their child about epilepsy at a higher rate than parents who were unemployed ($p = 0.026$) | Sample restricted to children with controlled epilepsy and no significant intellectual disabilities, learning difficulties, or developmental delay/ | CwE and their families need medical but therapeutic support to obtain an acceptable level of quality of life.<br><br>Physicians need to communicate effectively with the parents of CwE, based on the informational needs and the level of understanding of each individual patient or the family member.<br><br>Information needs to be delivered starting with the basics after the diagnosis, in a step-by-step approach, including additional sources addressing emotional issues at a personal or family level. Education materials need to be available.<br><br>Parents should be encouraged and informed about how to speak with other parents in similar situations.<br><br>Children with epilepsy and their families should be referred to experts for introducing them to helpful coping strategies, obtaining more information about the disorder, and diminishing fear and insecurity. | Not reported. |
| Maiga et al.<br>2014<br>Mali | To assess parental beliefs and attitudes in families with and without affected children. | **Design:** Quantitative descriptive.<br><br>**Methods:** verbal presentation of questionnaire-based survey<br><br>**Participants:** Parents. | **Recruitment:** Parents of children with epilepsy were systematically approached for enrolment during their child's regular consultation.<br>**Key eligibility:** Parent of child with epilepsy (0-15yrs) documented by the hospital's Department of Pediatrics or Neurology or both. | **Parents:** n=720 (n=360 epilepsy; n=360 control).<br>**Gender/role:** mother (almost 80%).<br>**Age:** epilepsy parents (mean 38.8 ± 8.9 yrs); control parents (34.1 ± 9.1 yrs).<br><br>**Children:** n=720<br>**Gender:** not reported.<br>**Age:** epilepsy children inpatients (mean 3.5 ± 2 yrs); control children (5 ± 3 yrs); epilepsy children outpatients (mean 6 ± 4 yrs); control children (7 ± 3 yrs). | **Diagnosis:** Epilepsy | Descriptive and inferential statistics. | **Fears related to retribution from the spirits:** some parents of CwE feared retribution for turning to modern medicine, or fear of a worsening of symptoms with the use of medication | Not reported. | **Fear of calamity:** linked to retribution from spirits. | Not reported. | Not applicable | Not reported. | Sample represent a relatively affluent subset of the population.<br><br>Findings very conservative, the general Malian population not having anywhere near the level of exposure to modern medicine that was evidenced in our sample (particularly the group with epilepsy). | Study highlights the urgent need to conduct widespread, intensified public education campaigns in the general population, as well as to open channels of communication and collaboration with traditional healers, to whom the population frequently turns for first-line care. | Not reported. |
| Murugupillai et al.<br>2016<br>Sri Lanka | To identify the concerns of parents regarding their children and adolescents with epilepsy in Sri Lanka | **Design:** Qualitative.<br><br>**Methods:** In-depth interviews and focus group discussion.<br><br>**Participants:** Parents, grandparents, and key informants (schoolteachers, public health staff) | **Recruitment:** Parents of children and adolescents (0-18yrs) with epilepsy identified from the paediatric and adult clinics in state hospitals in three districts.<br><br>**Key eligibility:** Parents of children with epilepsy, regardless of type and duration of epilepsy and included parents whose children had co-morbid illnesses (e.g., cerebral palsy). | **Parents:** n=16.<br>**Gender/role:** female (n=14); male (n=2).<br>**Age:** range 28-54yrs, mean (39yrs 9 months).<br><br>**Children:** n=16<br>**Gender:** female (n=5), male (n=11)<br>**Age:** range 5-18yrs, mean (12yrs 3 months). range yrs (mean yrs). | **Diagnosis:** Epilepsy.<br><br>**Seizure type:** simple partial (n=9), complex partial (n=2), generalised tonic-clonic (n=4), absence (n=1).<br><br>**Seizure frequency:** >1 per week (n=2), >1 per month (n=3), >1 per year (n=11).<br><br>**Age of seizure onset:** range 3 days-7yrs (mean 5yrs 11months).<br><br>**Medication:** Sodium valproate (n=15), Carbamazepne (n=2), Topiramate (n=1), Clobazam (n=2), Clonazepam (n=1).<br><br>**Number of anti epileptic drugs:** 1 drug (n=12), 2 drugs (n=3), 3 drugs (n=1).<br><br>**Comorbid illness:** (cerebral palsy) (n=3). | Content analysis. | **Fear about seizures:** Almost all participants expressed major distress about unexpected nature of occurrence of seizures.Fear about child having a seizure when alone.<br><br>**Fear associated with adverse effects of drugs:** Most parents worried about adverse effects that may occur with long-term use of anti-epileptic medicines.<br><br>**Fear of recurrence of seizures:** influences compliance in taking anti-epileptic medications.<br><br>**Fear of functional impact of epilepsy:** fear of epilepsy affecting child's physical ability to function normally.<br><br>**Fear associated with learning difficulties:** Parents concerned about epilepsy causing learning difficulties (e.g., problems in understanding, memorizing, slowness and laziness). | **Impact on social activities:** parents prevented children from bathing in the river, climbing trees, riding bicycles to avoid any catastrophic events.<br><br>**Impact of severe learning difficulties:** Parents worried that childish behaviour that may lead to exploitation and sexual abuse. And worries about providing necessary physical and mental support to manage activities of daily living of the child.<br><br>**Impact on school performance:** parents worried that epilepsy causing learning difficulties resulted in poor school performance. Daytime sedation adversely affecting schooling of the child was a concern.<br><br>**Impact of lack of trained teachers:** concerns that teachers would not understand and cope with the child.<br><br>**Impact on child's future:** worries related to possible difficulties in employment opportunities, marriage prospects and family life. | **Impact of negative comments:** parents worried about child being upset emotionally.<br><br>**Impact on personal image:** parents concerned that epilepsy would make their child sad and stressed and possibly develop a negative personal image.<br><br>**Impact on siblings:** some parents concerned about the problems in adjustment with siblings.<br><br>**Impact of stigma:** Most parents did not want to tell others about their child's epilepsy because of fear of stigma and social exclusion. Some people in community intimated to parents not to bring child to gatherings. | Not reported. | Not applicable. | Cost of treatment was unbearable to some families especially at times when they have to buy medicines outside the hospital pharmacy.<br><br>Cultural practices in the community influence the attitudes and behaviour of the parents. All of the parents reported that they sought religious/spiritual healing at least once. | Purposive sampling and small sample size may not represent all parents of children and adolescents with epilepsy in Sri Lanka.<br><br>Inclusion of parents of children and adolescents with epilepsy with co-morbid illnesses could have had a confounding effect on certain HRQL issues. | Consider whether parental concerns have same relative importance to children. | Future qualitative work with epilepsy patients can also investigate curious questions raised by this study (e.g., the relative importance of these parental concerns for patients). |

| | | | | | | | | | | | | | | | |
|---|---|---|---|---|---|---|---|---|---|---|---|---|---|---|---|
| Nguyen et al.<br>2015<br>Australia | To explore the cognitions and coping behaviours used by parents of children with epilepsy, in the aftermath of an epilepsy diagnosis, and the resultant effect on parental adjustment. | **Design:** Qualitative, realist approach.<br><br>**Methods:** Semi-structured interview.<br><br>**Participants:** Parents/ legal guardians. | **Recruitment:** Purposive sampling of biological parent or legal guardian of a child via the Neurology Department's database at the Women's and Children's Hospital in South Australia and via Epilepsy Centre of South Australia.<br><br>**Key eligibility:** parent of child (1-15yrs) diagnosed with epilepsy by a Consultant Neurologist 6 months - 5 years ago. | **Parents:** n=21.<br>**Gender/role:** mother(n=21); father (n=0);<br>**Age:** Mean 39.7yrs.<br><br>**Children:** n=21<br>**Gender:** female (n=9), male (n=12)<br>**Age:**not reported. | **Diagnosis:** Epilepsy.<br><br>**Age at diagnosis:** mean 6yrs, SD 3.82.<br><br>**Time since diagnosis:** mean 2.6yrs, SD 1.71.<br><br>**Type of seizure:** absence (n=5), tonic/clonic (n=4), myoclonic (n=1), simple partial (n=2), complex partial (n=4), mixture (n=5). | Deductive thematic analysis. | **Fear of seizures:** including fear of child not growing out of epilepsy / still needing medication in future. | **Impact on parental vigilance:** parents talked of being vigilant and then moderating vigilance. | **Impact on sense of control:** feeling helpless and vulnerable and loss of control (if unable to control seizures or if seizures worsen (e.g., absence to full blown seizure).<br><br>**Fear of future:** fear of how child will manage in the future, although noted this could be demoralizing and counter-productive. | Not applicable. | Not applicable. | Normalizing the impact of epilepsy on their child and lifestyle enabled parents to embrace a sense of normalcy into their lives.<br><br>Making comparisons with more aggressive or debilitating health conditions moved mothers to re-evaluate the gravity and perceive epilepsy management as comparatively easier.<br><br>Mothers maintained an optimistic outlook and chose to believe in the best case scenario of their child growing out of epilepsy in the near future.<br><br>Problem-solving, emotional venting, time to self and speaking with parents in similar situations were behaviours that buffered against carer strain. | Only mothers' perspectives reported.<br><br>Eight mothers had experience in nursing or in occupations that involved working with children with special needs.<br><br>A few children had co-morbid illness; no comparison possible between this group and non-comorbid group. | More opportunities needed for parents to converse with other role-model families should be facilitated.<br><br>An accessible network of contacts or support group can be a strong resource of experiential information and inspiration for parents confronted with newly diagnosed epilepsy.<br><br>Parents with significant psychological sequelae may require clinical interventions beyond standard psycho-education (e.g., cognitive re-structuring techniques, behavioural activation pro-gramme, emotional expressiveness, assertiveness training and problem solving skills). | Not reported. |
| O'Toole et al.<br>2016<br>Ireland | To explore the challenges that parents of children with epilepsy experience when engaging in dialog with their children about epilepsy and epilepsy-related issues. | **Design:** Qualitative.<br><br>**Methods:**. Interview (alone/together).<br><br>**Participants:** Parents. | **Recruitment:** via a tertiary referral children's neurology department (purposive) in a major children's hospital (n = 23) and also through a national epilepsy association.<br><br>**Key eligibility:** Parent/primary caregiver of a child (6-16yrs) with a diagnosis of epilepsy for ≥6 months and with a prescription for anti epileptic drugs at the time of interview. | **Parents:** n=34.<br>**Gender/role:** mother (n=27); father (n=7);<br>**Age:** not reported.<br><br>**Children:** not reported.<br>**Gender:** not reported<br>**Age:** range 6-16yrs (mean 11yrs 7months). | **Diagnosis:** Epilepsy.<br><br>**Age at diagnosis:** range 2yrs- 14yrs 6months (mean 7yrs 2months).<br><br>**Seizure type:** generalized tonic-clonic (n=18), absence (n=14), autonomic (n=7), dyscognitive (n=7), myoclonic (n= 5), atonic (n=2).<br><br>**Seizure frequency:** daily (n=7), weekly (n=4), monthly (=4), several times a year (n=8), once a year (n=6), seizure-free (n=1).<br><br>**Treatment path:** polytherapy (n=16), monotherapy (n=12), vagus nerve stimulation therapy (n=1), not currently receiving treatment (n=1). | Thematic analysis. | **Fear of misinforming child:** parents feared misinforming their child when faced with explaining aspects of epilepsy to their child because they were often the primary source of information for the child (e.g., talking about disclosure, growing out of epilepsy, seizure-freedom).<br><br>**Concern about accessing information:** parents often felt unsure of where to access reliable information specific to their child's diagnosis/seizure type and/or often found information they sourced difficult to understand . | Impacts on family - Parents report 'adjusting' to avoid making child feel different. But this does mean experiences for the siblings are different (i.e no sleepovers). | **Impact on child with epilepsy:** epilepsy-related communication may leave a child feeling 'singled out' in comparison to their siblings. Parents want to help child avoid feelings of differentness. | Not reported. | Not applicable. | Not reported. | Potential for sampling bias; most parents had child with refractory epilepsy and recruited via tertiary referral route, no significant comorbidities. | HCPs need to tailor their communication to each parent's need for specific information about their child's epilepsy diagnosis classification.<br><br>Enhanced parental understanding of their child's epilepsy condition could instill greater confidence in parents when conversing with their child about his/her condition.<br><br>Provision of child-friendly, epilepsy-related information for children living with epilepsy could facilitate greater ease of communication between parents and children | Not reported. |
| Ramachandra nnair et al.<br>2013<br>Canada | To understand the range of parental views on whether and how to approach the issue of SUDEP with families, to clarify the optimal timing and formulation of the information, and to learn from parents the optimal counseling strategies in order to minimize the inherent emotional burden. | **Design:** Qualitative descriptive.<br><br>**Methods: Focus groups, interviews.**<br><br>**Participants:** Parents. | **Recruitment:** Stratified purposeful sampling of SUDEP parents via clinic death registry and lay organisation and parents of children with epilepsy identified from the hospital epilepsy database and recruited via the Neurology Clinic at McMaster Children's Hospital (Ontario, Canada).<br><br>**Key eligibility:** Parents of children with mild, moderate to severe epilepsy, or new onset epilepsy epilepsy (≤17 yrs) or parents of children who had SUDEP. | **Parents:** n=42. Parents of children with epilepsy (n=36), SUDEP parents (n=6).<br>**Gender/role:** mother (n=21); father (n=15); not reported (n=6).<br>**Age:** not reported.<br><br>**Children:** children with epilepsy (n=21), SUDEP children (n=4).<br>**Gender:** not reported.<br>**Age:** not reported. | **Diagnosis:** Epilepsy or SUDEP.<br><br>**Seizure type:** new-onset epilepsy (=5), mild epilepsy (=9), moderate-severe epilepsy (n=7). | Directed content analysis. | **Fear of dying/SUDEP:** mothers and fathers highly concerned/worried child would die (either during a seizure or as a result of an injury during a seizure), fear especially focused on seizure and death at night. | **Impact on night-time routines:** Majority of parents said child slept in their bed as part of a coping strategy. Few parents adopted a nightly monitoring routine (e.g., therapy dog and monitors).<br><br>**Impact on parenting role:** fears of seizures resulted in parents being highly vigilant regarding their children and wanting to monitor all of their activities. | **Impact of child's diagnosis on mother:** mothers talked of a significant number of mental health issues: depression, anxiety, fear, worry, guilt, and exhaustion.<br><br>**Impact on father:** Fathers expressed feelings of worry, anger (directed toward the health care team), and concern for spouse and family.<br><br>**Impact of risk of SUDEP:** parents described feeling overwhelmed, worried, and increasingly anxious when the risk of SUDEP was explained to them but relieved when low risk of SUDEP explained. Fathers tended to express feeling uneasy, uncertain, and frustrated because of the lack of measures they could implement to prevent SUDEP. | Not specifically reported. | Not applicable. | Parents thought clear information about SUDEP at/soon after diagnosis and with follow-up information would be supportive. | Parents who participated may have been information seekers as opposed to information avoiders.<br><br>More than 90% of participants were Caucasians. | Optimal timing and setting of SUDEP counseling should certainly be determined on a case-by-case basis (by paediatric neurologist, typically early on and with sufficient time allotted, including realistic appraisal of risk and emphasis on prevalence and with follow-up and with printed materials), taking into account the degree of emotional and mental saturation that might currently exist in parents of newly diagnosed children. | Not reported |
| Rani et al.<br>2019<br>India | To assess parental knowledge, attitudes, and perceptions about epilepsy as well as addressing the socio-cultural barriers to its treatment. | **Design:** Mixed methods (descriptive).<br><br>**Methods:** questionnaire, clinical profile, scales, semi-structured interviews.<br><br>**Participants:** Parents. | **Recruitment:** Parents of children with epilepsy were recruited via neurology department of a tertiary referral centre.<br><br>**Key eligibility:** Parents of children with generalised or partial seizures, aged 4-15yrs; including children with a comorbid diagnosis ADHD, autism, intellectual development disorder, cerebral palsy | **Parents:** n=60.<br>**Gender/role:** mother (n=17); father (n=43).<br>**Age:** 25-35yrs (n=22), 36-45yrs (n=22), 46-55yrs (n=12);mean 37.2yrs.<br><br>**Children:** n=60<br>**Gender:** female (n=20), male (n=40)<br>**Age:** <6yrs (n=14), 6-10yrs (n=24), >10yrs (n=22); mean 8.4yrs. | **Diagnosis:** Epilepsy.<br><br>**Seizure type:** generalized seizures (n=30), partial seizures (n=19), combination of generalised and partial (n=11).<br><br>**Seizure control:** not controlled (n=44), controlled (n=16).<br><br>**Comorbid condition:** cerebral palsy (n=16), intellectual developmental delay (IDD) (n10), autism (n=2), ADHD (n=2). | **Quantitative:** descriptive statistics and non-parametric tests.<br><br>**Qualitative:** Process not described. | **Fears about seizures:** worries about seizures being fatal or life threatening<br><br>**Fears about medication:** worries about side-effects of medicine. | **Impact on social activities:** most parents prevented child from participating in sports activities.<br><br>**Impact on school:** most parents encouraged child to continue schooling despite epilepsy.<br><br>**Impact on parenting:** difficult to trust someone to care for the child. | **Impact on parental well-being:** parents generally worried.<br><br>**Impact of stigma:** parents apprehensive and concerned regarding disclosure of seizures.<br><br>**Impact on child:** parents believed children required more social and practical support for psycho-social development. | Not reported. | Not applicable. | Cultural issues mitigating factors (e.g., belief that epilepsy is a mental illness or caused by evil spirits). Many consulted faith-healers or holy men for treatment, visited holy places or performed 'mannat'. | Small cross-sectional sample, and time constraints, and sampling bias. Questionnaire developed to assess parents' knowledge, attitude, and perception not yet validated. | Mobilise support from different stakeholders who can act as change agents to sensitize the general public by addressing the myths and misconception related to epilepsy and community-based treatment and rehabilitation programs in rural areas<br><br>A management plan to address socio-cultural barriers to treatment should incorporate culturally sensitive practices by clinicians, understand parents' religious and spiritual values, be able to empathize, respect, and appreciate the efforts made by family members to cope with chronicity of the child's illness, sense of helplessness, and feelings of guilt for failing as a parent | Not reported. |
| Renardin et al.<br>2019<br>Brazil | To describe the perception and families living with childhood epilepsy | **Design:** Qualitative descriptive.<br><br>**Methods:** Semi-structured interviews.<br><br>**Participants:** Parents. | **Recruitment:** recruited via a doctor's office that specialised in neuropaediatrics.<br><br>**Key eligibility:** Parents of children with epilepsy, aged 5-12yrs, diagnosed ≥6 months. | **Parents:** n=7.<br>**Gender/role:** mother (n=6); father (n=1).<br>**Age:** range 25-42yrs (mean 35.8 yrs).<br><br>**Children:** n=7<br>**Gender:** not reported.<br>**Age:** range 6-11yrs 9months. | **Diagnosis:** Epilepsy | Thematic content analysis | **Fear for the future:** insecurity quality of life, learning, autonomy, and future of the child. | **Impact on parenting:** assuming defensive/ overprotective behaviours (e.g. rigorous supervision) towards the child.<br><br>**Impact on activities/participation:** restriction of activities aims to avoid crises.<br><br>**Impact on daily routine:** restructuring e daily routine according to demands imposed by the condition. | **Impact of insecurity and stigma:** concern about stigma and prejudice, can lead to the retraction of their child from social interaction.<br><br>**Impact on family well-being:** restructuring daily routine impacts on parental well-being (e.g., not sleeping, increased vigilance).<br><br>**Impact on parents:** parents experience fear, guilt, uncertainty, and despair, often motivated by negative stereotypes of epilepsy. | Not reported. | Not applicable. | Stigma. | Small sample size.<br><br>Study carried out in a single local specialty center. | Need to give voice to families in terms of their care demands, while seeking to contribute to professional practice, highlighting the important role of the nurse as an agent in the process of demystification of beliefs and qualification of health care. | Not reported. |
| Roberts et al.<br>2011<br>Canada | To improve understanding of the school experiences of children with epilepsy. | **Design:** Qualitative, Eidetic (descriptive) phenomenology.<br><br>**Methods:** In-depth, semi-structured interviews.<br><br>**Participants:** Caregivers. | **Recruitment:** not reported.<br>**Key eligibility:** Caregivers of children with epilepsy. | **Caregivers:** n=7.<br>**Gender/role:** not reported.<br>**Age:** not reported.<br><br>**Children:** n=7<br>**Gender:** not reported.<br>**Age:** range 5-11yrs. | **Diagnosis:** Epilepsy.<br><br>**Age of seizure onset:** 2.5-11yrs.<br><br>**Type of seizure:** absence (n=2), petit mal (n=1), grand mal (n=2), complex partial (n=2).<br><br>**Medication:** One child not medicated, Carbamazepine (n=1), Levetiracetam (n=1), Zarontin (n=1), Keppra (n=2), Lamictal and Epival (n=1). | Phenomenologic al. | **Fears about prognosis:** feelings of uncertainty about prognosis for their child and unpredictable nature of the condition.<br><br>**Fears of condition worsening:** many families concerned about the possibility of increased seizure severity.<br><br>**Fear about distinguishing symptoms:** some caregivers had difficulty in distinguishing symptoms of their child's epilepsy, and possible comorbid conditions. | **Impact on parenting:** loss of control over child's safety when sending them to school.<br><br>**Impact related to schooling:** beginning school after diagnosis was source of worry for caregivers. Some parents child was missing school due to 'medication errors'. Concerns re teachers lack of seizure first aid knowledge.<br><br>**Impact on learning:** some discussed the specific learning difficulties that their children experienced in the classroom. One child was home schooled.<br><br>**Impact on activities/participation:** need to place restrictions and limitations on activities the children could participate in. | **Impact of diagnosis:** experience of the diagnosis was emotional (stress, confusion, misunderstanding and uncertainty).<br><br>**Impact on caregivers:** continual worrying over the well-being of their child.And stress associated with understanding and explaining child's condition to the school community.<br><br>**Impact on parents:** parents often anxious when child at school, and some could not relate to other parents because of their child's epilepsy, and therefore did not feel a sense of belonging at their school.<br><br>**Impact of epilepsy of child's relationships:** some children experienced social isolation due to their epilepsy.<br><br>**Impact of stigma:** disclosure about diagnosis and medication changes elicited a fear that child would be treated differently. | Not specified. | Not reported. | Not reported. | None specified. | Not reported. | Not reported. |

| | | | | | | | | | | | | | | | |
|---|---|---|---|---|---|---|---|---|---|---|---|---|---|---|---|
| Saburi<br>2011<br>Zimbabwe | To identify stressors of caregivers of school-age children with epilepsy and to evaluate whether use of community resources alleviates or contributes to caregiver stress. | **Design:** Qualitative (descriptive cross-sectional).<br><br>**Methods:** Interviews.<br><br>**Participants:** Caregivers. | **Recruitment:** Convenience sample recruited from the ESF clinic at the George Nicholas Rehabilitation Center and from Parirenyatwa Central Hospital in Harare.<br><br>**Key eligibility:** Caregivers who have looked after child (6-17yrs) diagnosed with epilepsy for >1yr, and on AEDs. | **Parents:** n=46.<br>**Gender/role:** predominantly mothers.<br>**Age:** not reported.<br><br>**Children:** n=46<br>**Gender:** not reported.<br>**Age:** not reported. | **Diagnosis:** Epilepsy.<br><br>**Seizure type:** unknown by 35 caregivers.<br><br>**Mean duration from onset of seizures to diagnosis:** 13 months.<br><br>**Medication:** most children were either on phenobarbitone or carbamazepine.<br><br>**Epilepsy-related injuries:** Most (n=35) children had not experienced any epilepsy-related injuries in the past 12 months. | **Qualitative:** coding.<br><br>**Quantitative:** descriptive statistics. | **Fear about the future:** many caregivers feared child would live with them forever because of worry about seizures occurring in their absence.<br><br>**Fear of injury due to seizures:** most feared consequences of seizures included road traffic accidents, drowning, or burns. Other fears included physical disability from falls, death, cognitive impairment, sexual abuse, and dependency. | **Impact on child's future:** worry about child's ability to fend for self in caregivers' absence or death. Worries about marriage prospects<br><br>**Impact on education:** worries about possibilities of good education.<br><br>**Impact on strained relationships with extended family:** included fear of contagion to children, seizures being attributed to the maternal side, and accusations of sourcing seizures from traditional healers in exchange for supernatural powers.<br><br>**Impact on finance:** cost of tablets drained the scarce family finances and cost more than their salaries. | **Impact of child's seizures on caregivers:** The child's seizures caused deep pain and sadness for many caregivers.<br><br>**Impact on child's participation:** some children played alone, were laughed at by other children and were isolated because of the inability to speak; some children were dissuaded by their parents because of fear of contagion.<br><br>**Impact of stigma:** non-disclosure occurred to protect the child from physical and emotional hurt. | Not reported. | Not applicable. | Religious or worship groups helped to lighten the caregiver burden; accept the illness as God's will and get encouragement, advice, money, emotional support, holy water, and the ability to fend off evil spirits.<br><br>Counselling and belonging to epilepsy support groups. | Non probability sampling.<br><br>Unknown seizure types.<br><br>non-Shona speakers not represented, rural populations under-represented.<br><br>Interval from onset of epilepsy to diagnosis based on caregivers' self-report so may be misleading. | Healthcare providers should routinely assess the effect of seizures on caregivers, advocate for more male and extended family involvement in caregiving, and provide adequate information onside effects of drugs and on seizures.<br><br>Nurses in developed countries could incorporate religious activities among CAM interventions to reduce caregiver stress.<br><br>Spiritual faith healers should be encouraged to refer clients with epilepsy for drug therapy and counseling. | Not reported. |
| Smith et al.<br>2014<br>USA | To explore caregivers' perceptions of the care giving process at different time periods post epilepsy diagnosis | **Design:** Qualitative.<br><br>**Methods:**Focus groups.<br><br>**Participants:** Caregivers. Focus groups. | **Recruitment:** Purposive sample recruited from the only level 4 comprehensive epilepsy centre at an urban academic medical centre in an economically disadvantaged state.<br><br>**Key eligibility:** caregivers of youth (<18yrs) who had a confirmed diagnosis of epilepsy (e.g., ICD-9 345 codes) and resided within an 80-mile radius of the medical center. | **Caregivers:** n=19.<br>**Gender/role:** female (n=16); male (n=3); mothers (n=14), fathers (n=2), grandmother (n=1), grandfather (n=1), foster parent (n=1).<br>**Age:** 20-30yrs (n=2), 31-40yrs (n=8), 41-50yrs (n=6), ≥51yrs (n=3).<br><br>**Children:** n=19<br>**Gender:** not reported.<br>**Age:** 1-2yrs (n=1), 3-5yrs (n=4), 6-11yrs (n=12), 12-15yrs (n=1), 16-17yrs (n=1). | **Diagnosis:** Epilepsy; intractable epilepsy (n=13); controlled (n=6).<br><br>**Current medication (current):** (n=19). | Thematic analysis. | **Fears about seizure uncertainty:** even if provider's treatment plan is followed, seizures can occur.<br><br>**Fear about the future:** caregiver uncertainty about who would watch over their child (and who would look after them in olde age). | **Impact on parental role:** need to be vigilant with health-care providers, teachers, and other community service agencies because they were uninformed about epilepsy, seizures, and available resources.<br><br>**Impact on parenting:** role changed due to constant monitoring for seizures.<br><br>**Impact on activities/participation:** concerns about socialization. | **Impact of stigma:** concerns about stigmatisation. | Not reported. | Not applicable. | Length of time since diagnosis and complexity of condition. | Small sample size.<br><br>Recruitment from referral centre results in higher proportion of patients with severe epilepsy.<br><br>Predominance of white female caregivers, and majority of participants were married. | Future quantitative studies should be designed to examine caregiving process variables, as well as caregiver outcomes, and to develop caregiver-specific interventions that are tailored to the particular needs of these caregivers in their journey with epilepsy over time. | Not reported. |
| Webster<br>2017<br>UK | To explore how side effects impact on the meanings children and their parents ascribed to medications for epilepsy. | **Design:** Qualitative.<br><br>**Methods:** Semi-structured interviews and auto driven photo-elicitation interviews.<br><br>**Participants:** Parents and children. | **Recruitment:** via through seven UK based charities using adverts (websites, online forums, social media pages, newsletters.<br><br>**Key eligibility:** not reported. | **Parents:** n=28 (24 families).<br>**Gender/role:** mother (n=24); father (n=4).<br>**Age:** not reported.<br><br>**Children:** children with epilepsy (n=13), siblings (n=14).<br>**Gender of children with epilepsy:** female (n=4), male (n=9)<br>**Age of children with epilepsy:** range 5-13yrs. | **Diagnosis:** Epilepsy.<br><br>**Medication:** treated solely with AEDs (n=14), combination of medications and the ketogenic diet (n=10). | Constructivist grounded theory. | **Fears about side effects:** many parents concerned about the side effects of ongoing current treatment; particularly link with mental illness and potential mind-altering consequences of drug.<br><br>**Fear that medication not beneficial:** some parents had requested their child be changed to a different medication when treatment was deemed to be ineffective or when current side effects were not seen to be worth the benefits gained. | Change in parental role: role changed due to their constant monitoring for seizures | Not reported. | Not reported. | **Similarities:** Fears about epilepsy as threat to child's life.<br><br>**Differences:** Children were primarily concerned with the process of ingesting medications, whereas the parents focused on current side effects of [illegible] | Not reported. | Not reported. | Not reported. | Explore the ways in which children view their medications in order to understand their adherence to treatment regimens, as their perspectives are likely to differ to the views of adults |
| Webster<br>2019<br>UK | To explore parents' experiences of uncertainty resulting from having a child with epilepsy | **Design:** Qualitative.<br><br>**Methods:** Semi-structured interviews.<br><br>**Participants:** Parents. | **Recruitment:** via through seven UK based charities using adverts (websites, online forums, social media pages, newsletters.<br><br>**Key eligibility:** not reported. | **Parents:** n=27 (23 families).<br>**Gender/role:** mother (n=23); father (n=4).<br>**Age:** not reported.<br><br>**Children:** n=22<br>**Gender:** female (n=8), male (n=14)<br>**Age:** range 3-18yrs. | **Diagnosis:** Epilepsy.<br><br>**Seizure aetiologies:** most common included hypoxic-ischaemic encephalopathy (n=55), ischaemic stroke (n=41) and intracranial haemorrhage (n=31). | Constructivist grounded theory. | **Fear/concerns related to uncertainty/instability of symptoms:** unpredictability of condition seen as particularly hard aspect to deal with especially as they felt had unanswered questions from health professionals and were not fully informed about child's condition.<br><br>**Fear about seizures and SUDEP:** concern about SUDEP and how this may cut short their child's life.<br><br>**Fear about the future:** concerns about impact of condition on their child's future, possibility of a limited future.<br><br>**Fear about puberty:** fear of what might happen at puberty and how they child's epilepsy may change during this life stage. | **Impact on child's future:** related to job prospects, and child may never be able to drive. | Not reported. | Fathers' aligned with those of mothers. | Not applicable. | Not reported. | Not reported. | Not reported. | Not reported. |
| Webster<br>2020<br>UK | To detail the extent to which risk featured in children's and parents' descriptions of everyday life with the condition and whether their perceptions of risk aligned. | **Design:** Qualitative.<br><br>**Methods:** Group interviews, semi-structured interviews, photo-elicitation interviews.<br><br>**Participants:** Parents and children. | **Recruitment:** via through seven UK based charities using adverts (websites, online forums, social media pages, newsletters.<br><br>**Key eligibility:** not reported. | **Parents:** n=28 (24 families).<br>**Gender/role:** mother (n=24); father (n=4).<br>**Age:** not reported.<br><br>**Children:** children with epilepsy (n=13), siblings (n=14).<br>**Gender of children with epilepsy:** female (n=4), male (n=9).<br>**Gender of siblings:** female (n=9), male (n=5)<br>**Age of children with epilepsy:** range 5-13yrs.<br>**Age of siblings:** range 6-16yrs. | **Diagnosis:** Epilepsy. | Constructivist grounded theory. | **Fears associated with seizures/ SUDEP:** fears included status epilepticus and SUDEP.<br><br>**Fears associated with physical risks:** risk of incurring injuries during seizures. | **Impact on activities and participation:** fears association with risks requires closer supervision (e.g. swimming, heights, roads). | However, children,Äôs concerns did not always align with those of their parents; while parents focused on new and reconceptualised physical risks o the child, the children,Äôs primary preoccupation was with being stigmatised by their peers. | Not reported. | Parents focused on physical risks associated with epilepsy, but children more concerned about being stigmatised by their peers. | Perceptions of risk were influenced by both the child's age and their condition. | Not reported. | Not reported. | Parents' decision making regarding the communication of risks to children.<br><br>Determine whether children think about stigma in terms of risk |
| Wo et al.<br>2018<br>Malaysia | To explore the experiences of parents and their children,and to identify the needs and challenges faced by parents and children in childhood epilepsy care. | **Design:** Qualitative.<br><br>**Methods:** In-depth, semi-structured interviews.<br><br>**Participants:** Parents and children. | **Recruitment:** Parents were recruited from the paediatric (8-18yrs) and adult neurology (15-18yrs) clinics of a tertiary hospital in Malaysia.<br><br>**Key eligibility:** parents of children (8-18yrs), diagnosed ≥6 months, ≤4 seizures in the past 6 months. | **Parents:** n=15<br>**Gender/role:** mother (n=12); father (n=3).<br>**Age:** range 33-58yrs (mean 42.1, SD 5.6yrs).<br><br>**Children:** n=15<br>**Gender:** female (n=7), male (n=8)<br>**Age:** range 8-17yrs (mean 12.7, SD 1.7). | **Diagnosis:** Idiopathic generalized epilepsy (n=8), childhood/juvenile absent epilepsy (n=6), and focal epilepsy (n=1).<br><br>**Duration of epilepsy:** range 2-10yrs (mean 5, SD 3.5yrs) | Descriptive phenomenology approach and thematic analysis. | **Fear of death:** fear child would die in first seizure.<br><br>**Fear of unpredictability of symptoms:** fear that their child may have a seizure at any time still persisted, even several years after the diagnosis of epilepsy.<br><br>**Fear about the future:** worries about child's health, school performance, and future (whether they would be able to live independently and have equal rights for employment).<br><br>**Fears about cause of epilepsy:** some families also believed that epilepsy was a 'supernatural' disease, caused by bad spirits. | **Impact on parenting:** continuously watching out for any possible triggers of a seizure attack.<br><br>**Impact on child's social activities/ participation:** parents imposed restrictions on their child's physical and social activities (e.g., sleepovers, overseas trip), as they were afraid that their child may have a seizure, and no one would know how to care for their child.<br><br>**Impact on work and finance:** some parents were forced to give up their jobs to take care of their child with epilepsy. | **Impact of first seizure on parent:** negative emotional reactions parents were upset, shocked, and worried.<br><br>**Impact on parents:** some parents developed health or mental health issues.Some parents became anxious and depressed, because of the stress in caring for their child with epilepsy. Constantly worried.<br><br>**Impact of disclosure:** fear of stigmatisation, concern that their child's future would be affected, and worry that their child would not be treated equally by the school teacher<br><br>**Impact of blame:** some mothers blamed themselves for 'causing' child to have epilepsy; some in-laws blamed the mother for 'causing' child's epilepsy. | Not reported. | **Similarities:** views similar in following areas: physical functioning, academic achievement, and bullying.<br><br>**Differences:** views differed in emotional functioning, behavioral changes, and interpersonal relationships. | Parents coped with their problems using two different coping strategies: problem-focused coping and effective emotional-focused coping.<br><br>Continuity of care was discussed as beneficial. | Only recruited children with controlled seizures.<br><br>Imbalance of gender, ethnicity, and socioeconomic status (SES). | Not reported. | Not reported. |
