# Supplementary material for: Parents’/caregivers’ fears and concerns about their child’s epilepsy: A scoping review
Source: PLoS One. 2022 Sep 6;17(9):e0274001. doi: 10.1371/journal.pone.0274001 (PMC9447888; doi:10.1371/journal.pone.0274001)
Supplement: S6 Table — (PDF) [file pone.0274001.s006.pdf]

S6 Table: Mixed Methods Appraisal Tool (MMAT)

STUDY		SCREENING QUESTIONS		QUALITATIVE STUDIES				
First author	Year	Are there clear research questions/aims?	Do the collected data allow to address the research questions/aims?	Is the qualitative approach appropriate to answer the research question?	Are the qualitative data collection methods adequate to address the research question?	Are the findings adequately derived from the data?	Is the interpretation of results sufficiently substantiated by data?	Is there coherence between qualitative data sources, collection, analysis and interpretation?
Amjad et al.	2016	Yes	Yes	Yes	Yes	Yes	Can't tell	Can't tell
Benson et al.	2017	Yes	Yes	Yes	Yes	Yes	Yes	Yes
Fayed et al.	2021	Yes	Yes	Yes	Yes	Yes	Yes	Yes
Jensen et al.	2017	Yes	Can't tell	Yes	Can't tell	Yes	Yes	Can't tell
Jones et al.	2014	Yes	Yes	Yes	Yes	Yes	Yes	Yes
Jones et al.	2019	Yes	Yes	Yes	Yes	Yes	Yes	Yes
Kampra et al.	2017	Yes	Yes	Yes	Yes	Yes	Can't tell	Can't tell
Murugupillai et al.	2016	Yes	Yes	Yes	Yes	Yes	Yes	Yes
Nguyen et al.	2015	Yes	Yes	Yes	Yes	Yes	Yes	Yes
O'Toole et al.	2016	Yes	Yes	Yes	Yes	Yes	Yes	Yes
Ramachandrannair et al.	2013	Yes	Yes	Yes	Yes	Yes	Yes	Yes
Renardin et al.	2019	Yes	Can't tell	Yes	Can't tell	Can't tell	Yes	Can't tell
Roberts et al.	2011	Yes	Yes	Yes	Yes	Yes	Yes	Yes
Saburu	2011	Yes	Yes	Yes	Yes	Yes	Yes	Yes
Smith et al.	2014	Yes	Can't tell	Yes	Yes	Yes	Yes	Yes
Webster	2017	Can't tell	Yes	Yes	Yes	Yes	Yes	Yes
Webster	2019	Yes	Yes	Yes	Yes	Yes	Yes	Yes
Webster	2020	Can't tell	Yes	Yes	Yes	Yes	Yes	Yes
Wo et al.	2018	Yes	Yes	Yes	Yes	Yes	Yes	Yes

STUDY		SCREENING QUESTIONS		QUANTITATIVE DESCRIPTIVE STUDIES				
First author	Year	Are there clear research questions/aims?	Do the collected data allow to address the research questions/aims?	Is the sampling strategy relevant to address the research question?	Is the sample representative of the target population?	Are the measurements appropriate?	Is the risk of nonresponse bias low?	Is the statistical analysis appropriate to answer the research question?
Fowler et al.	2021	Yes	Yes	Yes	Can't tell	Yes	Can't tell	Yes
Gazibara et al.	2014	Yes	Yes	Yes	Yes	Yes	Can't tell	Yes
Maiga et al.	2014	Yes	Can't tell	Yes	Can't tell	Can't tell	No	Yes

STUDY		SCREENING QUESTIONS		MIXED METHODS STUDIES				
First author	Year	Are there clear research questions/aims?	Do the collected data allow to address the research questions/aims?	Is there an adequate rationale for using a mixed methods design to address the research question?	Are the different components of the study effectively integrated to answer the research question?	Are the outputs of the integration of qualitative and quantitative components adequately interpreted?	Are divergences and inconsistencies between quantitative and qualitative results adequately addressed?	Do the different components of the study adhere to the quality criteria of each tradition of the methods involved?
Benson et al.	2016	Yes	Yes	Yes	Yes	Yes	Can't tell	Can't tell
Rani et al.	2019	Yes	Yes	Can't tell	Can't tell	Can't tell	Can't tell	Yes
